# Proteomic Analysis of Various Rat Ocular Tissues after Ischemia–Reperfusion Injury and Possible Relevance to Acute Glaucoma

**DOI:** 10.3390/ijms18020334

**Published:** 2017-02-05

**Authors:** Hsin-Yi Chen, Hsiu-Chuan Chou, Shing-Jyh Chang, En-Chi Liao, Yi-Ting Tsai, Yu-Shan Wei, Ji-Min Li, Li-Hsun Lin, Meng-Wei Lin, Ying-Jen Chen, Yu-Sheng Chen, Chih-Chun Lin, Yi-Shiuan Wang, Mei-Lan Ko, Hong-Lin Chan

**Affiliations:** 1 Institute of Bioinformatics and Structural Biology & Department of Medical Sciences, National Tsing Hua University, Hsinchu 300, Taiwan; dful690@yahoo.com.tw (H.-Y.C.); nakyla1215@gmail.com (E.-C.L.); peach0722@hotmail.com.tw (Y.-T.T.); t91050127@hotmail.com.tw (Y.-S.W.); j77801@hotmail.com (J.-M.L.); lishlin0207@gmail.com (L.-H.L.); eva1018cat@yahoo.com.tw (M.-W.L.); chipmunk210@hotmail.com (Y.-J.C.); leo931016@yahoo.com.tw (Y.-S.C.); jennylin40728@yahoo.com.tw (C.-C.L.); woowoow0320@gmail.com (Y.-S.W.); 2 Department of Biomedical Engineering and Environmental Sciences, National Tsing Hua University, Hsinchu 300, Taiwan; chouhc@mail.nhcue.edu.tw; 3 Center for Teacher Education, National Tsing Hua University, Hsinchu 300, Taiwan; 4 Department of Applied Science, National Hsinchu University of Education, Hsinchu 300, Taiwan; 5 Gynecologic Oncology Section Department of Obstetrics and Gynecology, Hsinchu Mackay Memorial Hospital, Hsinchu 300, Taiwan; testisfine@gmail.com; 6 Department of Ophthalmology, National Taiwan University Hospital Hsin-Chu Branch, Hsinchu 300, Taiwan

**Keywords:** acute glaucoma, ischemia–reperfusion (IR) model, cornea, proteomics, difference gel electrophoresis (DIGE), matrix-assisted laser desorption/ionization time-of-flight (MALDI-TOF)

## Abstract

Glaucoma is a group of eye diseases that can cause vision loss and optical nerve damage. To investigate the protein expression alterations in various intraocular tissues (i.e., the cornea, conjunctiva, uvea, retina, and sclera) during ischemia–reperfusion (IR) injury, this study performed a proteomic analysis to qualitatively investigate such alterations resulting from acute glaucoma. The IR injury model combined with the proteomic analysis approach of two-dimensional difference gel electrophoresis (2D-DIGE) and matrix-assisted laser desorption/ionization time-of-flight mass spectrometry (MALDI-TOF MS) was used to monitor the protein expression alterations in two groups of specimens (an IR injury group and a control group). The analysis results revealed 221 unique differentially expressed proteins of a total of 1481 proteins in the cornea between the two groups. In addition, 97 of 1206 conjunctival proteins, 90 of 1354 uveal proteins, 61 of 1180 scleral proteins, and 37 of 1204 retinal proteins were differentially expressed. These findings imply that different ocular tissues have different tolerances against IR injury. To sum up, this study utilized the acute glaucoma model combined with 2D-DIGE and MALDI-TOF MS to investigate the IR injury affected protein expression on various ocular tissues, and based on the ratio of protein expression alterations, the alterations in the ocular tissues were in the following order: the cornea, conjunctiva, uvea, sclera, and retina.

## 1. Introduction

Acute glaucoma is a considerable cause of severe vision loss and irreversible blindness worldwide [1]. It is most commonly observed among people of Asian descent, partially because of their more crowded anterior chamber [2,3]. A rapid increase in intraocular pressure (IOP) to levels exceeding eye pressure results in eye ischemia with an increase in intracellular calcium levels and generation of reactive oxygen species, which have been shown to cause endoplasmic reticulum stress [4,5]. It is quite different from the type of chronic glaucoma, which cause progressive damage to the optic nerve and continuous loss of nerve fibers. The pathogenesis is distinct and the damage rate is slower compared with acute glaucoma.

Glaucoma, which is a group of eye diseases that can cause optical nerve damage, has been identified as one of the most crucial risk factors for cataracts [6]. Elevated IOP is the most crucial risk factor for glaucoma, and a sufficiently high IOP level can cause ocular tissue damage [7,8]. The study has reported that systemic hypertension or glaucoma might change the protein secondary structures of the immature cataractous lens capsule and alter ionic transport through the deformed lens capsule [9]. Therefore, protein expression alterations in various intraocular tissues, including the cornea, conjunctiva, uvea (composed of the iris, ciliary body, and choroid), retina, and sclera, during ischemia–reperfusion (IR) injury, may play a crucial role in clinical ophthalmology.

IR injury can be divided into ischemic injury and reperfusion injury. Ischemic injury results in a lack of blood supply in tissues or organs and energy shortage in cells, causing tissue damage. During tissue ischemia, which is caused by blocked blood flow, tissues do not receive oxygen or glucose, leading to reduced glycolysis and oxidative phosphorylation and reduced intracellular adenosine triphosphate (ATP) levels. Therefore, all energy-consuming reactions in the cells are affected [10]. The Na^+^/K^+^ ATPase pump and Ca^2+^ ATPase pump, which maintain the intracellular and extracellular ions and membrane potential balance, are impaired. The intracellular Na^+^ and Ca^2+^ concentrations increase, causing an elevated intracellular potential. Thus, some voltage-gated channels open to accelerate the attainment of the intracellular and extracellular membrane potential balance, and the intracellular membrane potential increases, which is called depolarization [11]. The elevation of the Ca^2+^ concentration is one of the indicative phenomena for adjacent cell injury during ischemia, and this elevated Ca^2+^ concentration can interfere with intracellular metabolism and lead to apoptosis through multiple pathways, and the free radicals produced in this process can further exacerbate the damage [12]. During reperfusion injury, rapid blood reperfusion into the ischemic tissue accelerates tissue damage. However, previous studies have not detected a large amount of free radicals in the tissue during ischemia, whereas a large number of free radicals are detected when the blood supply is restored to the ischemic tissue in a short time [13]. The restoration of blood supply can result in the accumulation of neutrophils in ischemic tissue, which can induce inflammation and release more free radicals [14]. Moreover, during ischemia, the overall activity of xanthine oxidase (XO) increases [15]. After restoration of the blood supply, the tissue receives sufficient oxygen to enable XO to catalyze hypoxanthine to form xanthine, and this reaction produces a large number of free radical by-products [16].

In the current proteomic studies, two-dimensional gel electrophoresis (2-DE) is a key technique for profiling thousands of proteins in biological samples, and this technique is complementary to liquid chromatography–mass spectrometry (LC/MS)-based proteomic analysis [17]. Nevertheless, reliable quantitative comparisons across gels and gel-to-gel variations reserve the major problem in 2-DE analysis. A crucial advance in 2D-gel-based protein detection and quantitation was accomplished with the application of two-dimensional difference gel electrophoresis (2D-DIGE), which can codetect multiple protein samples in the same 2-DE. This approach is able to reduce gel-to-gel variations and enables quantifying the relative abundance of protein characteristics across different gels through using a molecular weight/pI-matched internal fluorescent standard. Moreover, compared with conventional 2-DE, the 2D-DIGE technique has the advantages of a broader dynamic range, higher sensitivity, and greater reproducibility. This novel technology depends on the prelabeling of protein samples with three (Cy2, Cy3 and Cy5) fluorescent dyes before electrophoresis. Each dye has a unique fluorescent wavelength, permitting numerous experimental samples with an internal standard to separate on the same gel. The internal standard, which is a mixture of an equal amount of the experimental samples in combination of the normalization data and enhances statistical confidence in relative protein quantitation across gels [18,19,20].

In the current study, the IR injury model was used to mimic acute glaucoma in rat eyes. However, few reports have described protein expression alterations in various intraocular tissues (i.e., the cornea, conjunctiva, uvea, retina, and sclera) during IR injury. To study the protein expression alterations in these ocular tissues, proteomic analysis and the ratio of protein expression alterations was applied to identify the protein-level alterations to qualitatively investigate the protein expression alterations resulting from acute glaucoma.

## 2. Results

### 2.1. Analysis of Various Ocular Tissues Including Cornea, Conjunctiva, Uvea, Retina, and Sclera by 2D-DIGE and MALDI-TOF

**Figure 1:**
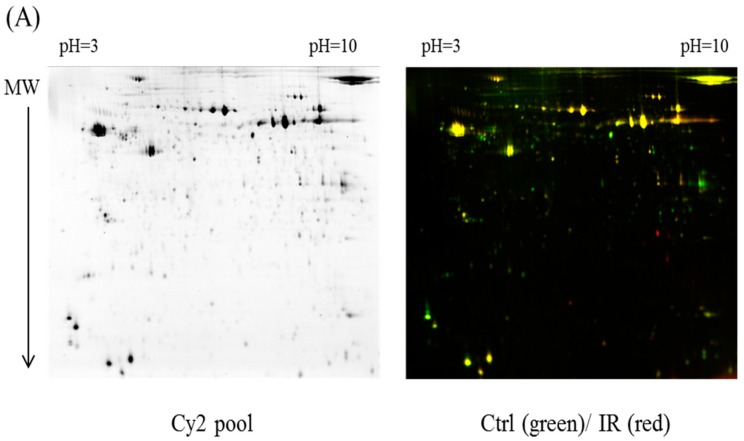

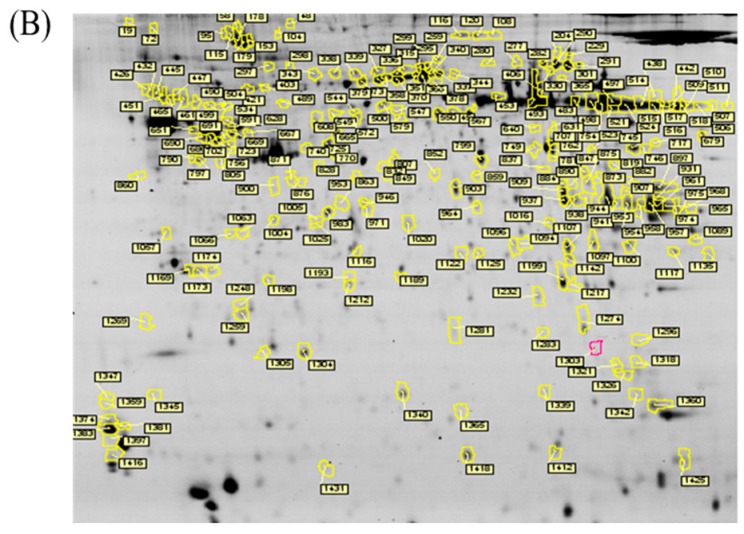
Comparison of proteomics between the ischemia–reperfusion (IR) injury and control groups (Ctrl) in cornea. MW: molecular weight (**A**) Schematic representation of the two-dimensional difference gel electrophoresis (2D-DIGE) workflow for monitoring the differentially expressed proteins in the cornea of the IR injury and control groups, and Cy2 pool represents the total protein spots; (**B**) 2D-DIGE images of the tissue samples analyzed using DeCyder software; the differentially expressed identified protein features are annotated with circles and spot numbers.

**Figure 2:**
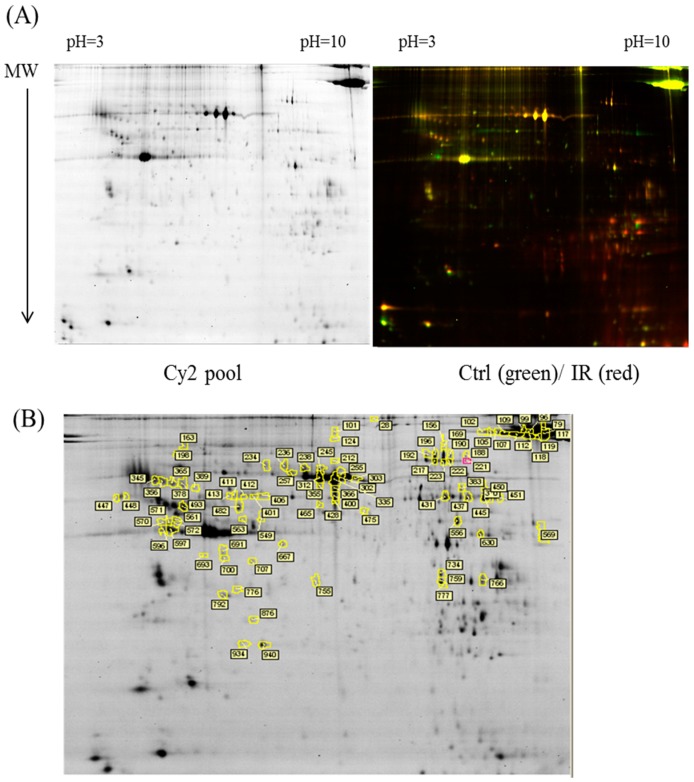
Comparison of proteomics between the IR injury and control groups in conjunctiva. (**A**) Schematic representation of the 2D-DIGE workflow for monitoring the differentially expressed proteins in the conjunctiva of the IR injury and control groups; (**B**) 2D-DIGE images of the tissue samples analyzed using DeCyder software; the differentially expressed identified protein features are annotated with circles and spot numbers.

**Figure 3:**
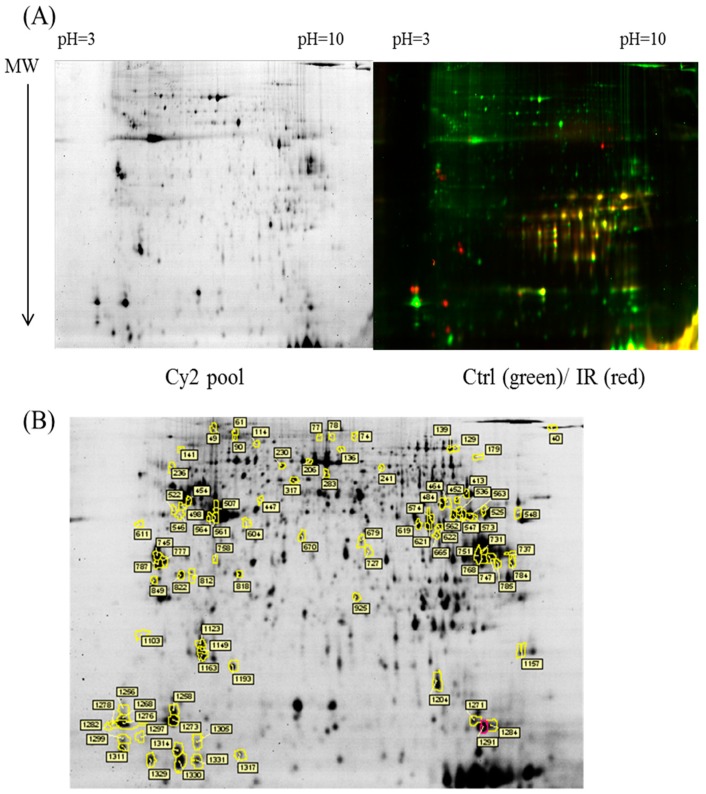
Comparison of proteomics between the IR injury and control groups in uvea. (**A**) Schematic representation of the 2D-DIGE workflow for monitoring the differentially expressed proteins in the uvea of the IR injury and control groups; (**B**) 2D-DIGE images of the tissue samples analyzed using DeCyder software; the differentially expressed identified protein features are annotated with circles and spot numbers.

**Figure 4:**
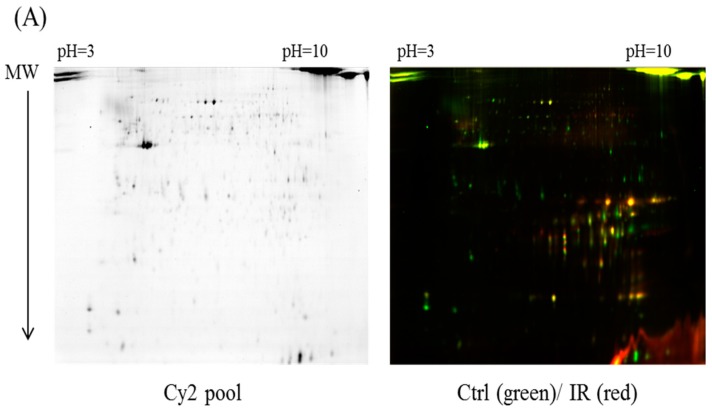

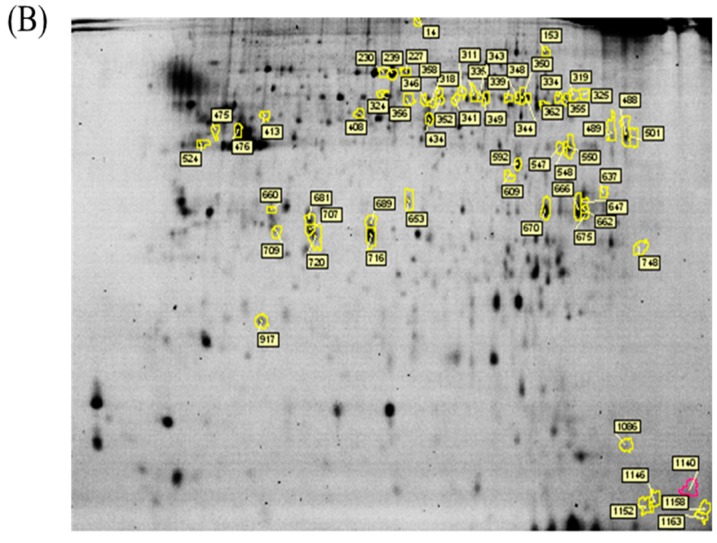
Comparison of proteomics between the IR injury and control groups in sclera. (**A**) Schematic representation of the 2D-DIGE workflow for monitoring the differentially expressed proteins in the sclera of the IR injury and control groups; (**B**) 2D-DIGE images of the tissue samples analyzed using DeCyder software; the differentially expressed identified protein features are annotated with circles and spot numbers.

**Figure 5:**
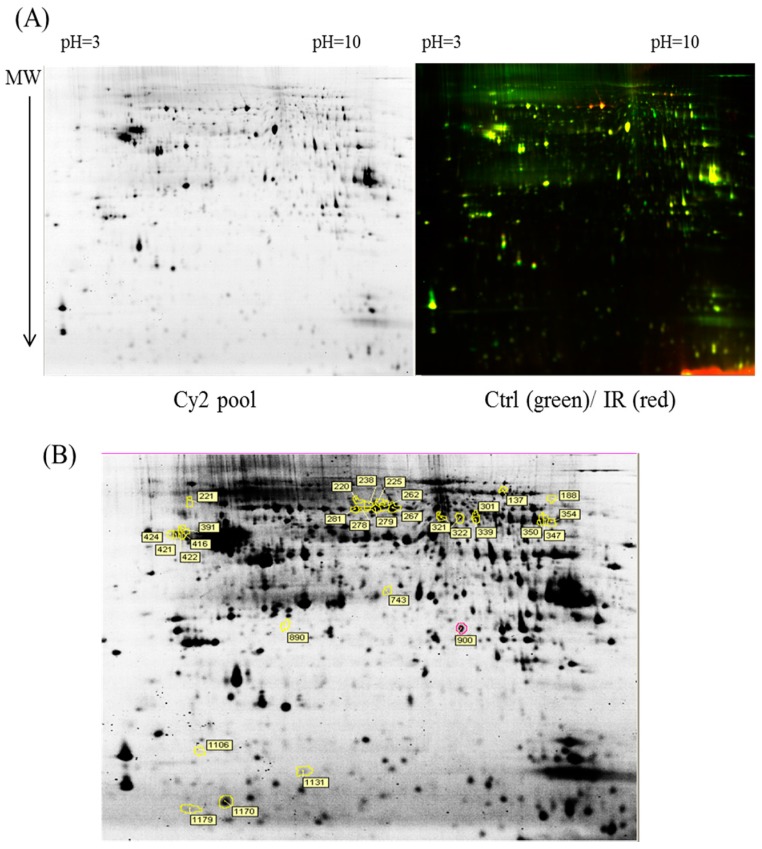
Comparison of proteomics between the IR injury and control groups in retina. (**A**) Schematic representation of the 2D-DIGE workflow for monitoring the differentially expressed proteins in the retina of the IR injury and control groups; (**B**) 2D-DIGE images of the tissue samples analyzed using DeCyder software; the differentially expressed identified protein features are annotated with circles and spot numbers.

**Figure 6:**
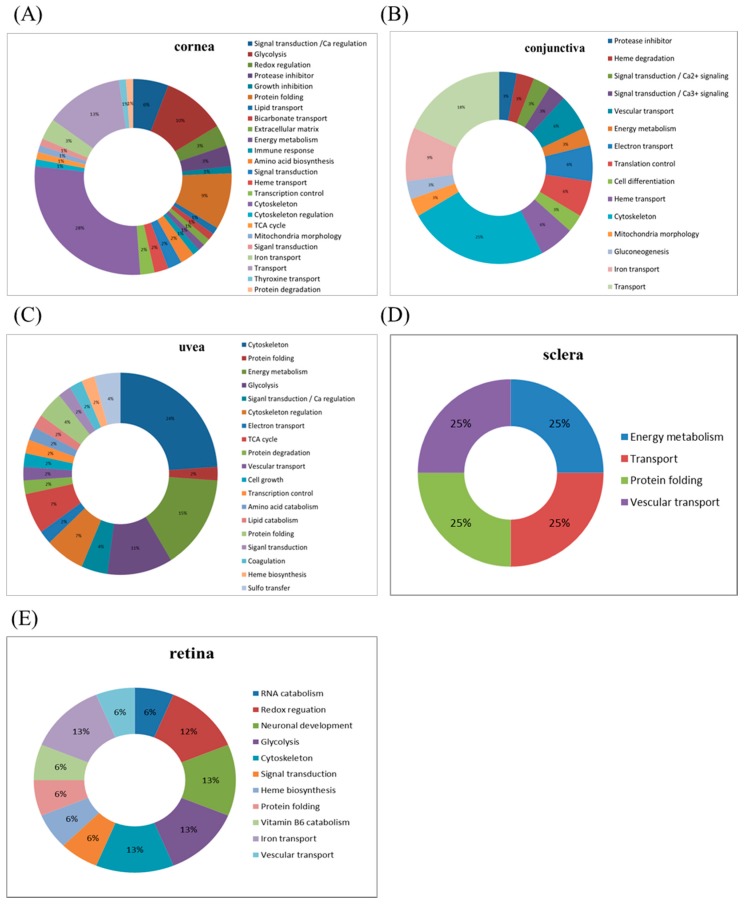
Percentage of total cellular proteins identified by 2D-DIGE/matrix-assisted laser desorption/ionization time-of-flight mass spectrometry (MALDI-TOF MS) for (**A**) cornea; (**B**) conjunctiva; (**C**) uvea; (**D**) sclera; and (**E**) retina according to their biological functions.

The dissected samples of various ocular tissues, including the cornea, iris, conjunctiva, retina, choroid, and sclera, with and without IR injury were labeled with CyDyes for 2D-DIGE analysis. Proteome profiling was visualized using a fluorescence scanner, and the images were superimposed using ImageQuant software (v7.0, GE Healthcare, Uppsala, Sweden). To investigate the protein expression alterations in various ocular cells in response to IR injury, DeCyder biological variation analysis was performed for protein spots showing more than a 1.3-fold change in expression with a Student *t*-test score less than 0.05; the images were visually inspected before confirming the alterations for protein identification (Figure 1, Figure 2, Figure 3, Figure 4 and Figure 5). It revealed 221 unique differentially expressed proteins of a total of 1481 proteins in the cornea between the control and IR injury groups. Moreover, 97 of 1206 conjunctival proteins, 90 of 1354 uveal proteins, 61 of 1180 scleral proteins, and 37 of 1204 retinal proteins were differentially expressed (Table 1). MALDI-TOF MS identified 86 cornea proteins, 33 conjunctiva proteins, 46 uvea proteins, 4 sclera proteins, and 16 retina proteins and combined with the search database showed the identified protein information (Supplementary Materials Tables S1–S5). The total cellular proteins identified by 2D-DIGE/MALDI-TOF MS for cornea, conjunctiva, uvea, sclera, and retina according to their biological functions (Figure 6).

### 2.2. Validation of Characterized Ocular Retinal Proteins through Immunoblotting

**Figure 7:**
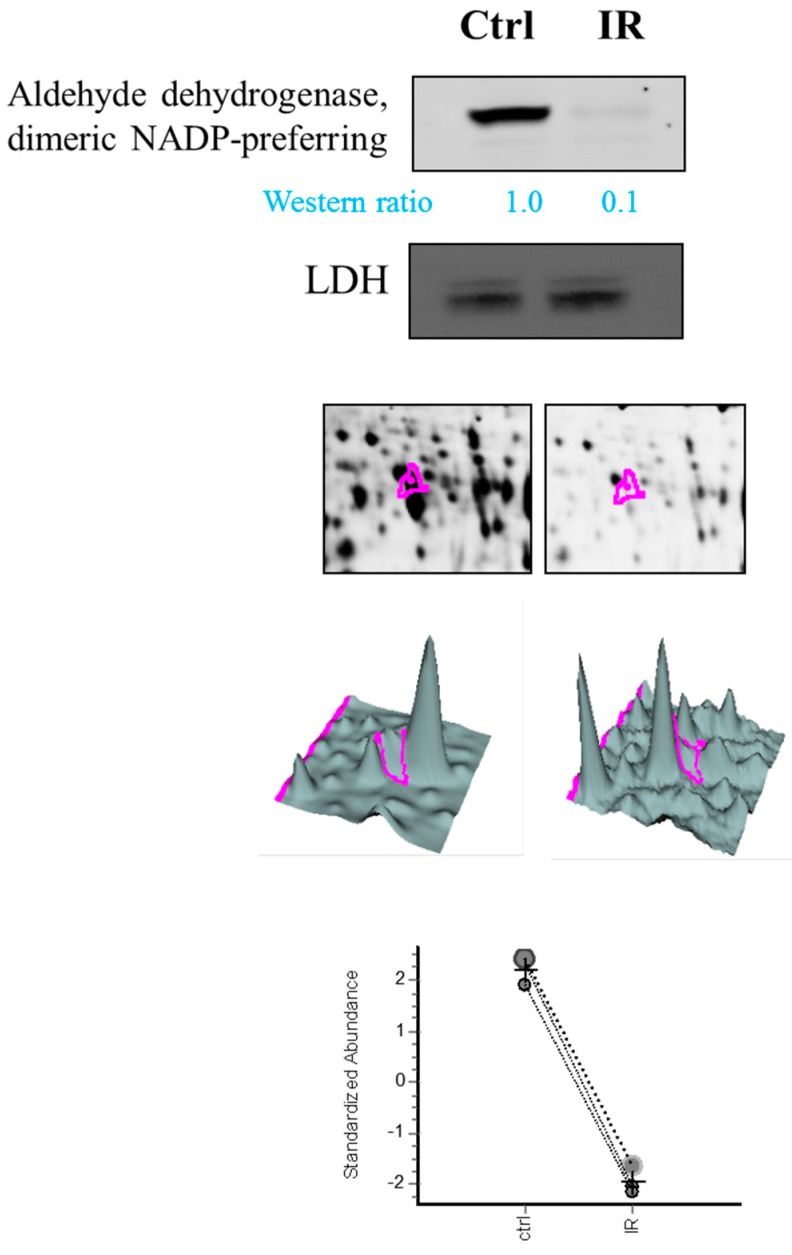
Representative immunoblotting analysis for selected differentially expressed protein identified by proteomic analysis between control groups and ischemia-reperfusion groups. The levels of identified proteins, Aldehyde dehydrogenase, dimeric dimeric nicotinamide adenine dinucleotide phosphate (NADP)-preferring, confirmed by immunoblot (top panels), with LDH (Lactate dehydrogenase) as loading controls. Protein expression map and three-dimension spot image are also shown. (The pink lines annotate the particular spot).

This glaucoma-based study identified some retinal cytosolic proteins such as aldehyde dehydrogenase (dimeric nicotinamide adenine dinucleotide phosphate (NADP)-preferring). It is essential to perform independent experiments to validate the expression level of these cytosolic proteins in the retina. Thus, the expression levels of aldehyde dehydrogenase (dimeric NADP-preferring), from the retina, were validated through immunoblotting. The results indicated that aldehyde dehydrogenase, dimeric NADP-preferring was down-regulated in ischemia-reperfusion groups and was consistent with the proteomic data from 2D-DIGE (Figure 7).

## 3. Discussion

This study used the platform of the acute glaucoma model combined with 2D-DIGE and MALDI-TOF MS and the ratio of protein expression alterations to investigate the influence of IR injury on various ocular tissues and to identify protein-level alterations. The IR injury model combined with proteomic analysis is sufficiently powerful for identifying protein expression alterations. The cornea contains various proteins and enzymes that contribute to its protective function, but it does not have blood vessels; it receives nutrients diffusion from the tear fluid and through the aqueous humor [21,22]. IR injury is induced by injecting saline into the anterior chamber, which may cause intraocular aqueous humor circulation and secretion or composition changes, thus directly leading to alteration in the expression of corneal cell proteins. An IOP higher than 80 mmHg due to IR injury may result in corneal endothelial cell dysfunction or even death owing to the changes in the physiological osmolality caused by the mechanical force. However, while the eye is open during IR injury induction, the corneal epithelial cells may partially receive oxygen from the atmosphere through dissolution in tears [23,24,25]; therefore, epithelial cell death may not occur despite high eye pressure. The protein expression alterations in the cornea are the most significant among those in other ocular tissues (cornea stroma and endothelium), implying that some unique mechanism occurs in the cornea in response to injuries such as IR injury. Based on the average ratio of protein expression alterations, crystalline proteins are upregulated during IR injury; their functional ontology is related to protein folding; other identified proteins (their roles are given in parentheses) are annexin A1/A2 (signal transduction and calcium regulation); hemopexin (heme transport); vitamin D-binding protein (transport); galectin-3 (immune response); and α-2-HS-glycoprotein (growth inhibition). By contrast, IR injury is associated with the downregulation of crystalline and proteins with functions in signal transduction, such as 14-3-3 gamma protein. The expression alterations mostly occur in proteins with functions in protein folding or signal transduction [26].

The conjunctiva lines the inner surface of the eyelids and covers the sclera. Histologically, the conjunctiva is composed of multilayered columnar epithelium and contains goblet cells. It can secrete mucus and tears to lubricate the eye, although the secretion of tears by the conjunctiva is less than that by the lacrimal gland. Moreover, the conjunctiva has the function of defense against microbial invasion [24]. IR models simulate severe environmental changes, and conjunctival defenses may be activated under such conditions, resulting in significant changes in protein expression. The mitochondrial proteins creatine kinase S-type, pyruvate carboxylase, and mitofusin-1 are upregulated in the conjunctiva. In addition, annexin A1 is upregulated in the cornea and downregulated in the conjunctiva.

The uvea is a layer of tissue below the white of the eye (sclera). It has the three following parts: the iris, which is the colored part of the eye; the choroid layer, which is the layer of blood vessels and connective tissue between the sclera and the retina; and the ciliary body, which secretes the aqueous humor into the eye. The choroid is a highly vascularized tissue accounting for approximately 90% of the intraocular blood flow. The tissue extends from the margins of the optic nerve head to the ora serrata. The choroid blood flow provides oxygen and nutrients to the outer retina and retinal pigment epithelium (RPE), which are the most metabolically active tissue in the body [24]. In our study, the expression alterations in the uvea were ranked third (after the cornea and then the conjunctiva). Therefore, the vascularized tissue of the uvea is first damaged, followed by the retina, which receives blood supply from the choriocapillaris. The protein expression alterations in the uvea are mostly related to proteins with functions in the cytoskeleton, energy metabolism, and glycolysis, such as myosin light chain 1/3, skeletal muscle isoform, parvalbumin alpha, tropomyosin, creatine kinase M-type, glyceraldehyde-3-phosphate dehydrogenase, which are upregulated. By contrast, destrin and calmodulin are downregulated owing to the characteristic of the uvea. Compared to other human research, there are 2815 kinds of proteins in the human ciliary body up to now, and most proteins are related to ubiquitin pathway, EIF2 signaling, glycogenolysis and gluconeogenesis, and aquaporin 1 (AQP1) [27].

The sclera is a fibrous connective tissue; the sclera in combination with IOP maintains the shape of the eyeball and the outer structure of the eye. The nerves and blood vessels that supply the nutrients inside the eye also pass through the sclera. Studies have indicated that protein extraction from the sclera is considerably difficult [28]. IR injury induced protein expression alterations, although the identification rate for the scleral proteins was low because of the ion signal of the sample. Creatine kinase M-type and heat shock cognate 71-kDa protein were found to be downregulated.

Numerous studies have shown data associated with retina that, astrocytes are associated with an increased inflammatory response to tumor necrosis factor-α/tumor necrosis factor receptor signaling, nuclear factor kappa-B activation, autophagy, and inflammasome expression [29]. In addition, these studies have demonstrated an increase in anticarbonyl reactivity and an increase in the carbonyl immunoreactivity of proteins such as HSP72 stress protein and glutamine synthetase [30]. Evidence also supports an increase in calpain in suicidal optic ganglion cells in the hypoxic monkey retina [31]. These findings demonstrate that neurodegeneration due to high IOP is associated with oxidative damage. However, in our study, the amount of protein expression alterations due to IR injury was relatively small in the retina compared with other tissues, presumably because its tolerance is comparable to that of other tissues. 

Notably, aldehyde dehydrogenase, dimeric NADP-preferring (ALDH3A1) is a member of the ALDH superfamily of proteins that catalyze the NAD(P)-dependent oxidation of a wide range of endogenous and exogenous aldehydes [32]. It plays a major role in the detoxification of alcohol-derived acetaldehyde and involved in the metabolism of corticosteroids, biogenic amines, neurotransmitters, and lipid peroxidation. It had been reported with a role in preventing corneal damage caused by ultraviolet light [33] and also resonates with the anti-apoptotic and cell growth regulating roles of the lens α-crystallins [34,35]. Despite the downregulated expression of ALDH3A1 in retina tissue, we also noted dihydropyrimidinase-related protein 2, protein disulfide-isomerase A3, Pyridoxal phosphate phosphatase, which are all upregulated during IR model. They are associated with neuronal development, protein folding, vitamin B6 catabolism, respectively. This result suggests specific roles of ALDH3A1 in IR model compared to other expression alteration proteins in retina.

In our study, the IR injury groups received ischemia for 1 h and reperfusion for 1 h. The purpose of this study was to explore the immediate change in vivo; if the duration of reperfusion is prolonged, perhaps the organization of the mechanism of recovery will change the amount of alterations.

The eyeball is the most complex organ in the body [36]. Despite its small size, it is a remarkable organ comprising ocular tissues with distinct functions and operation mechanisms. The oxidative stress produced by IR injury results in severe and mild damage in various ocular tissues. The ratio of protein expression alterations in our study was based on the IR injury model, in which a 30-gauge needle connected to a saline reservoir is inserted into the anterior chamber, but not the vitreous. The model should be modified for needle insertion into the vitreous, and additional studies should verify our results.

## 4. Materials and Methods

### 4.1. Animals

The experimental procedures were approved by the Animal Ethics Committee of National Tsing Hua University (10460, 25 December 2015). Animals were treated in accordance with the Association for Research in Vision and the Ophthalmology Statement for the Use of Animals in Ophthalmic and Vision Research. Sprague–Dawley rats aged 7–8 weeks were divided into two groups: a control group and an IR injury group (6 per group). The IR group received ischemia for 1 h followed by reperfusion for 1 h. The rats were housed in an environment with a 12-h light/dark cycle and were provided ad libitum access to standard chow and water. The ischemia procedure, tissue dissection, and tissue incubation were performed under normal room illumination.

### 4.2. IR Injury Model

The rats were deeply anesthetized by intraperitoneal injection with a 1:1 mixture of ketamine (0.5 mg/mL, Pfizer, New York, NY, USA) and xylazine (20 mg/mL, Bayer, Leverkusen, Germany) at a dose of 0.32 mL per 200 g body weight. The heads of the rats were stabilized in a stereotaxic frame. One drop of proparacaine hydrochloride (0.5%, Alcon Lab, Hünenberg, Switzerland) was instilled into the cornea to ensure complete anesthesia; subsequently, a 30-gauge needle connected to a saline reservoir was inserted into the anterior chamber of the left eye. The saline reservoir was elevated to a height of 2 m, and the valve was opened to induce retinal ischemia for 1 h (IOP of 80 mmHg). Ischemia was confirmed by observing the whitening of the iris and loss of the red reflex. For the control group, a 30-gauge needle was inserted into contralateral right eye with cannulated saline reservoir, but no elevation. After ischemia induction, the rats were placed in cages for reperfusion. After 1 h of reperfusion, the eyeball was enucleated. Various ocular tissues (e.g., the cornea, iris, conjunctiva, retina, choroid, and sclera) were dissected and stored at −80 °C for further proteomic analysis.

All rats were deeply anesthetized using a 1:1 mixture of overdoses (2 mL per 200 g body weight) of ketamine and xylazine and were euthanized with CO_2_ in a box.

### 4.3. Chemicals and Reagents

General chemicals were obtained from Sigma-Aldrich (St. Louis, MO, USA); additionally, the reagents and dyes for 2D-DIGE were purchased from GE Healthcare (Uppsala, Sweden). All primary antibodies were obtained from Genetex (Hsinchu, Taiwan) and the secondary antibodies were obtained from GE Healthcare (Uppsala, Sweden). All biochemicals and chemicals used in this research were of analytical grade.

### 4.4. 2D-DIGE and Gel Image Analysis

Before performing 2D-DIGE, the various ocular tissues were solubilized in 2D-DIGE lysis buffer (4% *w*/*v* 3-((3-cholamidopropyl)dimethylammonio)-1-propanesulfonate (CHAPS), 7 M urea, 2 M thiourea, 10 mM Tris–HCl (pH 8.3), and 1 mM EDTA). Samples were homogenized by passage through a 26-gauge needle 20 times, insoluble material was removed by centrifugation at 13,000 rpm for 30 min at 4 °C, and protein concentrations were determined using Bradford Coomassie Protein Assay Reagent (BioRad, Irvine, CA, USA).

Protein samples were labeled with *N*-hydroxy succinimidyl ester-derivatives of the cyanine dyes of Cy2, Cy3 and Cy5 following a previously described protocol [37,38]. Accordingly, protein sample was minimally labeled with Cy3 or Cy5 dyes with a ratio of 2.5 pmol dye per microgram of for comparison in the same 2-DE. To accelerate image comparison as well as cross-gel statistical quantification, a mix of all protein samples was also prepared and labeled with fluorescent Cy2 at a molar ratio as the same as Cy3 and Cy5 dyes in this study as an internal standard across all gels. Therefore, triplicate protein samples with the internal standard could be resolved and quantified via multiple 2-DE. The labeling process was carried out in the dark on ice for 0.5 h and then stopped the reaction by quenching with a 20-fold molar ratio excess of free l-lysine to dye for another 10 min. The differentially Cy3- and Cy5-labeled samples were then mixed with the Cy2-labeled internal standard and reduced with dithiothreitol. Nonlinear immobilized pH gradient (IPG) buffer (pH 3–10, 2% (*v*/*v*), GE Healthcare) was added, and the final volume was adjusted to 450 μL by using 2D lysis buffer for rehydration. In the rehydration process, 24-cm nonlinear IPG strips (pH 3–10) were used to rehydrate CyDye-labeled samples in the dark at room temperature overnight (at least 12 h). Isoelectric focusing was then performed using a Multiphor II apparatus (GE Healthcare) for a total of 62.5 kVh at 20 °C. Strips were equilibrated in 6 M urea, 30% (*v*/*v*) glycerol, 1% (*w*/*v*) sodium dodecyl sulfate (SDS), 65 mM dithiothreitol, and 100 mM Tris–HCl (pH 8.8) for 15 min followed by the same buffer containing 240 mM iodoacetamide for another 15 min. The equilibrated IPG strips were then transferred onto 12.5% polyacrylamide gels (26 × 20 cm^2^) casted between two glass plates with low-fluorescence property. The equilibrated IPG strips were overlaid with low-melting point agarose (0.5% (*w*/*v*)) in a running buffer containing 0.01% of bromophenol blue. The 2D gels were run at 4 W/gel at 10 °C in an Ettan Twelve gel tank (GE Healthcare) until the bromophenol blue dye front had entirely run off the bottom of the gels. Afterwards, the fluorescent 2-DE gels were scanned straightly between the low-fluorescence glass plates through an Ettan DIGE Imager which is a charge-coupled device enables scanning the gels with Cy2-, Cy3- and Cy5-wavelengths. (GE Healthcare). Gel analysis was performed based on DeCyder Software version 7.0 (GE Healthcare) with the abilities of codetection, normalization, and quantification of the protein features in the images. The substances monitored from non-protein contaminants were filtered out by the software. Protein spots with an average ≥1.3-fold increase or decrease in abundance as well as with a *p* value of <0.05 were selected for further protein identification.

### 4.5. Protein Staining

Colloidal coomassie blue G-250 (CCB) staining was performed to imagine CyDye-labeled protein features in 2-DE. Briefly, gels were fixed in 30% (*v*/*v*) ethanol and 2% (*v*/*v*) phosphoric acid for at least 3 h, followed by washed with ddH_2_O for three times (30 min each), and then incubated in 34% (*v*/*v*) methanol, 17% (*w*/*v*) ammonium sulfate, and 3% (*v*/*v*) phosphoric acid for another 1 h before adding 0.5 g/L CCB. The gels were then stained for 5–7 days. No destaining step was required for this staining strategy. The stained gels were then visualized through ImageScanner III densitometer (GE Healthcare), which generated the gel images in TIF format.

### 4.6. In-Gel Digestion

Picked poststained gel pieces were washed two times in 50% acetonitrile plus 50% ammonium bicarbonate, followed by reduced with 5 mM ammonium bicarbonate containing 10 mM dithiothreitol (pH 8.0) for 45 min at 50 °C, and then alkylated the reduced proteins with 5 mM ammonium bicarbonate containing 50 mM iodoacetamide for 1 h at room temperature in the dark. The gel pieces were subsequently washed two times in 50% acetonitrile and vacuum-dried before reswelling with 10 mM ammonium bicarbonate containing 50 ng of modified trypsin (Promega, Madison, WI, USA). The pieces were then overlaid with 10 µL of 10 mM ammonium bicarbonate and performed trypsinization for 16 h at 37 °C. Supernatants were collected for peptide extraction at least twice with 50% acetonitrile containing 1% trifluoroacetic acid, and the supernatants were pooled for vacuum-dried, and stored at −20 °C before MS analysis.

### 4.7. Protein Identification by MALDI-TOF MS

The extracted proteins were cleaved using a proteolytic enzyme to generate peptides. Subsequently, a peptide mass fingerprint (PMF) database was searched, followed by MALDI-TOF MS for protein identification. In brief, 0.5 µL of a tryptic-digested peptide sample was mixed with 0.5 µL of a matrix solution containing *R*-cyano-4-hydroxycinammic acid (1 mg *R*-cyano-4-hydroxycinammic acid in 1 mL of 50% acetonitrile (*v*/*v*)/0.1% trifluoroacetic acid (*v*/*v*)), spotted onto an anchorchip target plate (Bruker Daltonics, Billerica, MA, USA), and waited for dry. The PMFs were obtained using an Autoflex III mass spectrometer (Bruker Daltonics) based on the reflector mode. The algorithm used for spectrum annotation was based on the process called sophisticated numerical annotation procedure (SNAP). This process used the following detailed parameters: Peak detection algorithm, SNAP; relative intensity threshold, 0%; signal-to-noise threshold, 25; maximal number of peaks, 50; quality factor threshold, 1000; minimum intensity threshold, 0; baseline subtraction, median; SNAP average composition, averaging; median level, 0.5; and flatness, 0.8. The spectrometer was calibrated with a peptide calibration standard (Bruker Daltonics). In addition, the trypsin autolysis peaks at *m*/*z* 842.51 and *m*/*z* 2211.10 were used for internal calibration as well. Peaks in the mass range of *m*/*z* 800–3000 were used to produce PMF data that were searched against the Swiss-Prot/TrEMBL database (version 2016_07) with 551705 entries by using Mascot software version 2.6.0 (Matrix Science, London, UK). The parameters used for the search: Rattus; carbamidomethylation of cysteine, tryptic digest with a maximum of 1 missed cleavage; partial modification of glutamine to pyroglutamate; partial methionine oxidation, and partial protein N-terminal acetylation; and a mass tolerance of 50 ppm. Identification was approved based on spectrum annotation, significant MASCOT Mowse scores (*p* < 0.05), observed versus expected molecular weight, and pI on 2-DE.

### 4.8. Immunoblotting

Immunoblotting was used to validate the differential expression of MS-identified proteins. Samples were lysed with 2D-DIGE lysis buffer (4% *w*/*v* CHAPS, 7 M urea, 2 M thiourea, 10 mM Tris–HCl (pH 8.3), and 1 mM EDTA) before protein quantification with the Coomassie Protein Assay Reagent (BioRad) and were consequently separated on 1D-SDS-PAGE. After electroblotting the gel-separated proteins onto 0.45-μm PVDF membranes (Millipore, Taipei, Taiwan) which were blocked with 5% (*w*/*v*) skim milk in TBST (50 mM Tris (pH 8.0), 150 mM NaCl, and 0.1% Tween-20 (*v*/*v*)) for 1 h followed by incubated with primary antibody solution in TBS-T containing 0.02% (*w*/*v*) sodium azide for 2 h. The membranes were washed at least three times in TBS-T for 10 min each and then probed with the corresponding horseradish peroxidase-coupled secondary antibody (GE Healthcare). After additional washing in TBS-T, the immunoblotted proteins were visualized using enhanced chemiluminescence strategy (Visual Protein Co., Taipei, Taiwan).

## 5. Conclusions

To sum up, this study utilized the acute glaucoma model combined with 2D-DIGE and MALDI-TOF MS to investigate the IR injury affected protein expression on various ocular tissues, and based on the ratio of protein expression alterations, the alterations in the ocular tissues were in the following order: the cornea, conjunctiva, uvea, sclera, and retina. Accordingly, pilot studies are recommended to use this proteomic strategy for the comprehensive analysis of protein-level alterations in various ocular tissues in order to provide valuable information for future investigations of the eye in health and disease.

## Figures and Tables

**Table 1:** Order of protein expression alterations (i.e., cornea, conjunctiva, uvea, sclera, and retina) according to the ratio of protein expression alterations.

Ocular Tissues	≥1.3 or ≤−1.3 (Fold Change)	Ratio (%)	Rankings
cornea	221/1481	14.92%	1
conjunctiva	97/1206	8.04%	2
uvea	90/1354	6.65%	3
sclera	61/1180	5.17%	4
retina	37/1204	2.49%	5

## Supplementary Materials

The following are available online at www.mdpi.com/1422-0067/18/2/334/s1.

## Abbreviations

1-DE	one-dimensional gel electrophoresis
2-DE	two-dimensional gel electrophoresis
Ab	antibody
CCB	colloidal coomassie blue
CHAPS	3-((3-cholamidopropyl)-dimethylammonio)-1-propanesulfonate)
ddH_2_O	double deionized water
DIGE	differential gel electrophoresis
DTT	dithiothreitol
MALDI-TOF MS	matrix assisted laser desorption ionization-time of flight mass spectrometry
TFA	trifluoroacetic acid
