# Supplementary material for: Proteomic Analysis of Various Rat Ocular Tissues after Ischemia–Reperfusion Injury and Possible Relevance to Acute Glaucoma
Source: Int J Mol Sci. 2017 Feb 5;18(2):334. doi: 10.3390/ijms18020334 (PMC5343869; doi:10.3390/ijms18020334)
Supplement: Supplementary file 1 [file ijms-18-00334-s001.pdf]

Supplementary Materials: Proteomic Analysis of Various Rat Ocular Tissues after Ischemia–Reperfusion Injury and Possible Relevance to Acute Glaucoma

Hsin-Yi Chen, Hsiu-Chuan Chou, Shing-Jyh Chang, En-Chi Liao, Yi-Ting Tsai, Yu-Shan Wei, Ji-Min Li, Li-Hsun Lin, Meng-Wei Lin, Ying-Jen Chen, Yu-Sheng Chen, Chih-Chun Lin, Yi-Shiuan Wang, Mei-Lan Ko and Hong-Lin Chan

Table S1. Alphabetical list of identified differentially expressed cornea proteins between the IR injury and control groups after 2D-DIGE coupled with MALDI-TOF mass spectrometry analysis. (pI: Isoelectric point; MW: Molecular weight; Cov.: Protein sequence coverage (%)).

Master No.	Swiss-Prot No.	Protein Name	pI	MW	Cov. (%)	Score	Matched Peptides	Subcellular Location	Functional Ontology	IR/Ctrl Av. Ratio	IR/Ctrl *t*-Test	No. Match Peptides
1169	P61983	14-3-3 protein γ	4.8	28456	26%	94/52	8/13	Cytoplasm	Signal transduction /Ca regulation	−1.32	0.0062	M.VDREQLVQK.A + Acetyl (Protein N-term) R.LAEQAER.Y K.NVTELNEPLSNEER.N
847	P51635	Alcohol dehydrogenase (NADP(+))	6.84	36711	19%	105/52	6/10	Cytoplasm	Glycolysis	−1.31	0.0034	M.TASSVLLHTGQK.M + Acetyl (Protein N-term) K.YALSVGYR.H K.HHPEDVEPAVR.K
406	P11883	Aldehyde dehydrogenase, dimeric NADP-preferring	6.33	50706	19%	142/52	8/16	Cytoplasm	Redox regulation	1.31	0.0021	R.IQQLEALQR.M K.SISGALASDLGK.N K.HLTPVTLELGGK.S
453	P11883	Aldehyde dehydrogenase, dimeric NADP-preferring	6.33	50706	26%	147/52	12/20	Cytoplasm	Redox regulation	1.31	0.023	M.SSISDTVKR.A + Acetyl (Protein N-term) R.IQQLEALQR.M R.FDHIMYTGSTAVGK.I + Oxidation (M)
493	P11883	Aldehyde dehydrogenase, dimeric NADP-preferring	6.33	50706	33%	164/52	16/31	Cytoplasm	Redox regulation	1.32	0.00015	M.SSISDTVKR.A + Acetyl (Protein N-term) R.IQQLEALQR.M K.ELPDWAEDEPVAK.T
651	P17475	α-1-antiproteinase	5.7	46278	10%	88/52	5/6	Secreted	Protease inhibitor	1.46	0.013	K.RPFNPEHTR.D R.DADFHVDK.S R.SAILYFPK.L
669	P17475	α-1-antiproteinase	5.7	46278	8%	67/52	4/6	Secreted	Protease inhibitor	1.54	0.0054	K.RPFNPEHTR.D R.SAILYFPK.L K.TLLSSLGITR.V
499	P24090	α-2-HS-glycoprotein	6.05	38757	15%	76/52	4/6	Secreted	Growth inhibition	1.37	0.0017	K.HLLQGFR.Q K.QYGFCK.A R.HAFSPVASVESASGEVLHSPK.V
1283	P24623	α-crystallin A chain	6.35	22490	16%	88/52	5/9	Cytoplasm	Protein folding	1.58	0.041	R.ALGPFYPSR.L R.QSLFR.T R.QDDHGYISR.E

Table S1. *Cont.*

Master No.	Swiss-Prot No.	Protein Name	pI	MW	Cov. (%)	Score	Matched Peptides	Subcellular Location	Functional Ontology	IR/Ctrl Av. Ratio	IR/Ctrl *t*-Test	No. Match Peptides
1340	P24623	α-crystallin A chain	6.35	22490	41%	169/52	13/23	Cytoplasm	Protein folding	2.59	0.00012	MDVTIQHPWFK.R + Acetyl (Protein N-term); Oxidation (M)<br>R.ALGPFYPSR.L<br>R.QSLFR.T + Gln->pyro-Glu (N-term Q)
567	P04764	α-enolase	6.16	47440	34%	175/52	14/24	Cytoplasm	Glycolysis	−1.93	8.8×10^{-6}	R.AAVPSGASTGIYEALELR.D K.AVEHINK.T<br>K.TIAPALVSK.K
th909	P07150	Annexin A1	6.97	39147	34%	149/52	11/15	Plasma membrane	Signal transduction /Ca regulation	1.38	0.022	M.AMVSEFLK.Q + Acetyl<br>(Protein N-term); Oxidation<br>(M) K.GGPGSAVSPYPSFNPSSDVAALHK.A<br>K.AAYLQETGKPLDETLKK.A
941	Q07936	Annexin A2	7.55	38939	39%	155/52	15/20	Plasma membrane	Signal transduction /Ca regulation	1.5	0.002	M.STVHEILCK.L + Acetyl (Protein N-term)<br>K.LSLEGDHSTPPSAYGSVKPYTNFDAER.D<br>R.QDIAFAYQR.R + Gln->pyro-Glu (N-term Q)
1212	P04639	Apolipoprotein A-I	5.52	30100	12%	65/52	4/7	Secreted	Lipid transport	1.59	0.00042	K.ETDWLR.N<br>K.VVAEEFR.D<br>K.FGLYSDQMR.E + Oxidation (M)
1281	P56374	β-crystallin A4	5.9	22596	47%	147/52	7/11	Cytoplasm	Protein folding	−1.31	0.039	K.SAGHWR.V R.VVVWDEEGFQGR.R<br>R.GDYPGWDAWGGNTAYPAER.L
1107	P02523	β-crystallin B1	6.84	28303	30%	97/52	6/19	Cytoplasm	Protein folding	−1.53	0.0018	K.GTPSTGTAPAPGPTPVPASVPRPAAK.V<br>K.VGELPPGSYR.L R.LVVFEQENFQGR.R
1274	P0C5E9	β-crystallin S	6.95	21327	39%	199/52	10/13	Cytoplasm	Protein folding	8.6	7.6×10^{-5}	K.ISFYEDR.N<br>R.NFQGR.R<br>R.SYLSR.C
1383	P62161	Calmodulin	4.09	16827	35%	83/52	5/8	Cytoplasm	Signal transduction/Ca regulation	−2.07	0.0031	M.ADQLTEEQIAEFK.E +<br>Acetyl (Protein N-term)<br>K.EAFSLFDKDGDGTITTK.E K.ELGTVMR.S +<br>Oxidation<br>(M)
1174	P47728	Calretinin	4.94	31499	14%	74/52	4/5	Cytoplasm	Signal transduction/Ca regulation	−4.41	0.032	K.ANRPYDEPK.L K.LQEYTQTILR.M<br>K.LGLSEMSR.L +<br>Oxidation (M)
1097	P27139	Carbonic anhydrase 2	6.89	29267	31%	139/52	9/17	Cytoplasm	Bicarbonate transport	−6.46	0.00012	M.SHHWGYSK.S<br>M.SHHWGYSK.S + Acetyl (Protein N-term)<br>K.SNGPENWHK.E
964	P02466	Collagen α-2(I) chain	9.39	129999	5%	65/52	8/17	Secreted	Extracellular matrix	−1.35	0.0037	R.GHNGLDGLK.G<br>R.SQPSLRPK.D<br>K.SLNNQIETLLTPEGSR.K

Table S1. *Cont.*

Master No.	Swiss-Prot No.	Protein Name	pI	MW	Cov. (%)	Score	Matched Peptides	Subcellular Location	Functional Ontology	IR/Ctrl Av. Ratio	IR/Ctrl *t*-Test	No. Match Peptides
725	P07335	Creatine kinase B-type	5.39	42983	30%	143/52	10/22	Cytoplasm	Energy metabolism	−3	0.00077	M.PFSNSHNTQK.L<br>K.VLTPELYAELR.A<br>K.DLFDPIIEDR.H
897	P05065	Fructose-bisphosphate aldolase A	8.31	39783	21%	106/52	6/17	Cytoplasm	Glycolysis	−1.42	0.00071	M.PHPYPALTPEQK.K<br>K.ELADIAHR.I<br>R.QLLLTADDR.V
1117	P08699	Galectin-3	8.59	27241	24%	90/52	7/10	Secreted	Immune response	1.31	0.00025	K.GNDIAFHFNPR.F<br>R.FNENNRR.V<br>K.QDNNWGR.E + Gln->pyro-Glu (N-term Q)
1326	P10066	Γ-crystallin B	7.55	21531	41%	141/52	9/22	Cytoplasm	Protein folding	4.55	0.025	K.ITFFEDR.G<br>R.GFQGR.C<br>R.CYECSSDCPNLQTYFSR.C
1321	P10067	Γ-crystallin D	6.99	21489	39%	133/52	9/10	Cytoplasm	Protein folding	6.19	0.00028	K.ITFYEDR.G<br>R.HYECSTDHSNLQPYFSR.C<br>R.LIPHAGSHR.I
1318	P10068	Γ-crystallin F	7.14	21670	50%	214/52	15/20	Cytoplasm	Protein folding	7.83	0.00094	M.GKITFYEDR.G<br>K.ITFYEDR.G<br>R.GFQGR.H
723	P07323	Γ-enolase	5.03	47510	14%	81/52	5/10	Cytoplasm	Glycolysis	−6.4	7.9×10^{-7}	R.AAVPSGASTGIYEALELR.D<br>R.LGAEVYHTLK.G<br>K.MVIGMDVAASEFYR.D + 2 Oxidation (M)
749	P09606	Glutamine synthetase	6.64	42982	8%	56/52	4/15	Cytoplasm	Amino acid biosynthesis	−2.06	0.012	R.KPAETNLR.H<br>R.DIVEAHYR.A<br>R.MGDHLWVAR.F + Oxidation (M)
762	P09606	Glutamine synthetase	6.64	42982	22%	148/52	9/12	Cytoplasm	Amino acid biosynthesis	−4.21	8.7×10^{-5}	K.LVFCEVFK.Y<br>R.KPAETNLR.H<br>R.DIVEAHYR.A
961	P04797	Glyceraldehyde-3-phosphate dehydrogenase	8.14	36090	31%	139/52	8/18	Cytoplasm	Glycolysis	−1.63	0.00057	K.VGVNGFGR.I<br>R.VIISAPSADAPMFVMGVNHEK.Y + 2 Oxidation (M)<br>K.IVSNASCTTNCLAPLAK.V
954	P04797	Glyceraldehyde-3-phosphate dehydrogenase	8.14	36090	15%	76/52	4/6	Cytoplasm	Glycolysis	−1.57	2.8×10^{-5}	R.GAAQNIIPASTGAAK.A K.LTGMAFR.V + Oxidation(M)<br>R.VPTPNVSVVDLTCR.L
957	P04797	Glyceraldehyde-3-phosphate dehydrogenase	8.14	36090	35%	163/52	10/19	Cytoplasm	Glycolysis	−1.44	0.0007	K.VGVNGFGR.I K.LVINGKPITIFQER.D<br>R.VIISAPSADAPMFVMGVNHEK.Y + Oxidation (M)

Table S1. *Cont.*

Master No.	Swiss-Prot No.	Protein Name	pI	MW	Cov. (%)	Score	Matched Peptides	Subcellular Location	Functional Ontology	IR/Ctrl Av. Ratio	IR/Ctrl *t*-Test	No. Match Peptides
1100	P04797	Glyceraldehyde-3-phosphate dehydrogenase	8.14	36090	10%	52/52	3/6	Cytoplasm	Glycolysis	−2.31	0.01	K.LTGMAFR.V + Oxidation (M)<br>R.VPTPNVSVVDLTCR.L<br>K.LISWYDNEYGYSNR.V
115	P63219	Guanine nucleotide-binding protein G(I)/G(S)/G(O) subunit γ-5	9.9	7428	26%	52/52	3/11	Plasma membrane	Signal transduction	1.46	0.0071	MSGSSSVAAMKK.V<br>M.SGSSSVAAMKK.V<br>K.KVVQQLR.L
983	P54311	Guanine nucleotide-binding protein G(I)/G(S)/G(T) subunit β-1	5.6	38151	25%	136/52	10/15	Plasma membrane	Signal transduction	−5.36	5.4×10^{-7}	M.SELDQLR.Q + Acetyl (Protein N-term)<br>R.KACADATLSQITNNIDPVGR.I<br>K.ACADATLSQITNNIDPVGR.I
338	P20059	Hemopexin	7.58	52060	12%	84/52	5/8	Secreted	Heme transport	1.49	0.023	K.NPVTSVDAAFR.G<br>R.GECQSEGVLFFQGNR.K<br>R.FNPVTGEVPPR.Y
339	P20059	Hemopexin	7.58	52060	22%	132/52	9/21	Secreted	Heme transport	1.57	0.0071	K.NPVTSVDAAFR.G<br>R.GPDSVFLIK.E<br>R.GECQSEGVLFFQGNR.K
974	A7VJC2	Heterogeneous nuclear ribonucleoproteins A2/B1	8.97	37512	27%	90/52	7/33	Nucleus	Transcription control	−1.57	0.0061	K.IDTIEIITDR.Q<br>R.GFGFVTFDDHDPVDK.I<br>K.YHTINGHNAEVR.K
975	A7VJC2	Heterogeneous nuclear ribonucleoproteins A2/B1	8.97	37512	24%	108/52	6/13	Nucleus	Transcription control	−1.61	0.037	K.IDTIEIITDR.Q<br>R.GFGFVTFDDHDPVDK.I<br>R.GGNFGFGDSR.G
790	Q6IFV3	Keratin, type I cytoskeletal 15	4.8	49011	11%	58/52	4/10	Cytoplasm	Cytoskeleton	−1.34	0.019	M.ATTFLQTSSTFGSGSTR.G + Acetyl (Protein Nterm)<br>R.VGGGSFGGGSLYGGGGSR.S<br>R.LAADDFR.L
797	Q6IFV3	Keratin, type I cytoskeletal 15	4.8	49011	24%	141/52	10/13	Cytoplasm	Cytoskeleton	−1.31	0.028	M.ATTFLQTSSTFGSGSTR.G + Acetyl (Protein Nterm)<br>R.VGGGSFGGGSLYGGGGSR.S<br>K.VTMQNLNDR.L + Oxidation (M)
517	Q6P6Q2	Keratin, type II cytoskeletal 5	7.6	61959	18%	155/52	9/12	Cytoplasm	Cytoskeleton	1.94	0.0081	R.QSSVSFR.S<br>R.SFSAASAITPSVSR.T<br>R.TTFSSVSR.S
523	Q6P6Q2	Keratin, type II cytoskeletal 5	7.6	61959	43%	313/52	26/48	Cytoplasm	Cytoskeleton	1.9	3.9×10^{-5}	R.QSSVSFR.S<br>R.SFSAASAITPSVSR.T<br>R.TTFSSVSR.S

Table S1. *Cont.*

Master No.	Swiss-Prot No.	Protein Name	pI	MW	Cov. (%)	Score	Matched Peptides	Subcellular Location	Functional Ontology	IR/Ctrl Av. Ratio	IR/Ctrl *t*-Test	No. Match Peptides
483	Q6P6Q2	Keratin, type II cytoskeletal 5	7.6	61959	27%	205/52	17/26	Cytoplasm	Cytoskeleton	1.43	0.001	R.QSSVSFR.S R.SFSAASAITPSVSR.T R.TTFSSVSR.S
497	Q6P6Q2	Keratin, type II cytoskeletal 5	7.6	61959	16%	168/52	10/12	Cytoplasm	Cytoskeleton	1.91	0.00021	R.QSSVSFR.S R.SFSAASAITPSVSR.T R.TTFSSVSR.S
498	Q6P6Q2	Keratin, type II cytoskeletal 5	7.6	61959	24%	189/52	13/25	Cytoplasm	Cytoskeleton	1.43	0.0016	R.QSSVSFR.S R.SFSAASAITPSVSR.T R.TTFSSVSR.S
500	Q6P6Q2	Keratin, type II cytoskeletal 5	7.6	61959	11%	103/52	7/11	Cytoplasm	Cytoskeleton	−1.55	0.0025	R.VSLGGAYGAGGYGSR.S R.ISFSSGGGSFR.N K.FASFIDK.V
506	Q6P6Q2	Keratin, type II cytoskeletal 5	7.6	61959	9%	87/52	5/6	Cytoplasm	Cytoskeleton	1.83	9.4×10^{-5}	R.QSSVSFR.S R.SFSAASAITPSVSR.T R.VSLGGAYGAGGYGSR.S
507	Q6P6Q2	Keratin, type II cytoskeletal 5	7.6	61959	14%	115/52	8/14	Cytoplasm	Cytoskeleton	1.63	0.025	R.SFSAASAITPSVSR.T R.VSLGGAYGAGGYGSR.S R.SLYNVGGSK.R
509	Q6P6Q2	Keratin, type II cytoskeletal 5	7.6	61959	24%	231/52	15/21	Cytoplasm	Cytoskeleton	1.84	0.00017	R.QSSVSFR.S R.SFSAASAITPSVSR.T R.TTFSSVSR.S
510	Q6P6Q2	Keratin, type II cytoskeletal 5	7.6	61959	20%	203/52	12/14	Cytoplasm	Cytoskeleton	2	0.0002	R.QSSVSFR.S R.SFSAASAITPSVSR.T R.TTFSSVSR.S
511	Q6P6Q2	Keratin, type II cytoskeletal 5	7.6	61959	22%	196/52	12/17	Cytoplasm	Cytoskeleton	1.68	0.024	R.QSSVSFR.S R.SFSAASAITPSVSR.T R.TTFSSVSR.S
514	Q6P6Q2	Keratin, type II cytoskeletal 5	7.6	61959	24%	152/52	11/24	Cytoplasm	Cytoskeleton	1.77	0.001	R.QSSVSFR.S R.SFSAASAITPSVSR.T R.VSLGGAYGAGGYGSR.S
515	Q6P6Q2	Keratin, type II cytoskeletal 5	7.6	61959	29%	197/52	17/41	Cytoplasm	Cytoskeleton	1.82	0.00024	R.QSSVSFR.S R.SFSAASAITPSVSR.T R.TTFSSVSR.S
516	Q6P6Q2	Keratin, type II cytoskeletal 5	7.6	61959	20%	196/52	12/15	Cytoplasm	Cytoskeleton	1.84	4.7×10^{-5}	R.QSSVSFR.S R.SFSAASAITPSVSR.T R.TTFSSVSR.S

Table S1. *Cont.*

Master No.	Swiss-Prot No.	Protein Name	pI	MW	Cov. (%)	Score	Matched Peptides	Subcellular Location	Functional Ontology	IR/Ctrl Av. Ratio	IR/Ctrl *t*-Test	No. Match Peptides
518	Q6P6Q2	Keratin, type II cytoskeletal 5	7.6	61959	12%	112/52	7/11	Cytoplasm	Cytoskeleton	1.95	3.3×10^{-5}	R.QSSVSFR.S<br>R.SFSAASAITPSVSR.T<br>R.TTFSSVSR.S
665	Q6P6Q2	Keratin, type II cytoskeletal 5	7.6	61959	15%	138/52	10/17	Cytoplasm	Cytoskeleton	−2.45	3.0×10^{-6}	K.FASFIDK.V<br>R.FLEQQNK.V<br>K.WTLLQEQGTK.T
770	Q6P6Q2	Keratin, type II cytoskeletal 5	7.6	61959	18%	127/52	11/26	Cytoplasm	Cytoskeleton	1.39	0.0017	K.FASFIDK.V<br>R.FLEQQNK.V<br>K.WTLLQEQGTK.T
799	Q6AYC4	Macrophage-capping protein	6.11	39060	16%	89/52	7/10	Cytoplasm	Cytoskeleton regulation	1.31	0.00015	K.LKPVPIAR.E<br>K.YREGGVESAFHK.T<br>R.EGGVESAFHK.T
903	O88989	Malate dehydrogenase, cytoplasmic	6.16	36631	11%	65/52	4/7	Mitochondria	TCA cycle	−1.33	0.011	K.ENFSCLTR.L<br>K.GEFITTVQQR.G<br>K.AISDHIR.D
805	Q8R4Z9	Mitofusin-1	6.11	84649	5%	54/52	5/7	Mitochondria	Mitochondria morphology	1.37	0.021	K.HFVLAKK.A<br>R.CLHFLVEELK.V<br>K.VVSPLEAR.N
1304	P31044	Phosphatidylethanolamine-binding protein 1	5.48	20902	39%	157/52	10/18	Plasma membrane	Siganl transduction	−1.47	0.00018	R.VDYGGVTVDELGK.V<br>K.LYTLVLTDPDAPSR.K<br>K.GNDISSGTVLSEYVGSGPPK.D
745	P16617	Phosphoglycerate kinase 1	8.02	44909	11%	61/52	5/8	Cytoplasm	Glycolysis	−1.53	5.0×10^{-5}	R.VDFNVPMK.N + Oxidation (M)<br>K.NNQITNNQR.I<br>K.LGDVYVNDAFGTAHR.A
465	P05544	Serine protease inhibitor A3L	5.48	46419	12%	54/52	5/21	Secreted	Protease inhibitor	1.42	0.00068	K.IAELFSDLEER.T R.TSMVLVNYLLFK.G + Oxidation (M)<br>K.FSISTDYSLK.E
282	P12346	Serotransferrin	7.14	78512	17%	203/52	13/17	Secreted	Iron transport	1.48	0.0034	K.WCAVSEHENTK.C<br>K.TVLPADGPR.L<br>K.GTDFQLNQLQGK.K
290	P12346	Serotransferrin	7.14	78512	29%	285/52	19/28	Secreted	Iron transport	1.52	0.00047	K.WCAVSEHENTK.C<br>K.TVLPADGPR.L<br>K.SCHTGLGR.S
301	P12346	Serotransferrin	7.14	78512	21%	190/52	13/19	Secreted	Iron transport	1.53	0.00046	K.TVLPADGPR.L<br>K.HTTIFEVLPQK.A<br>R.KPVDQYEDCYLAR.I

Table S1. *Cont.*

Master No.	Swiss-Prot No.	Protein Name	pI	MW	Cov. (%)	Score	Matched Peptides	Subcellular Location	Functional Ontology	IR/Ctrl Av. Ratio	IR/Ctrl *t*-Test	No. Match Peptides
295	P02770	Serum albumin	6.09	70682	29%	180/52	16/25	Secreted	Transport	1.64	0.013	R.FKDLGEQHFK.G<br>K.DLGEQHFK.G<br>K.GLVLIAFSQYLQK.C
299	P02770	Serum albumin	6.09	70682	46%	255/52	27/53	Secreted	Transport	1.61	0.001	R.EAHKSEIAHR.F<br>K.SEIAHRFK.D<br>R.FKDLGEQHFK.G
336	P02770	Serum albumin	6.09	70682	9%	59/52	5/8	Secreted	Transport	1.55	0.0052	K.CPYEEHIK.L<br>K.LVQEVTDFAK.T<br>K.SIHTLFGDK.L
337	P02770	Serum albumin	6.09	70682	12%	73/52	5/6	Secreted	Transport	1.59	0.0019	K.GLVLIAFSQYLQK.C<br>K.LGEYGFQNAVLVR.Y<br>K.APQVSTPTLVEAAR.N
351	P02770	Serum albumin	6.09	70682	31%	212/52	20/35	Secreted	Transport	1.57	0.012	R.EAHKSEIAHR.F<br>R.FKDLGEQHFK.G<br>K.DLGEQHFK.G
358	P02770	Serum albumin	6.09	70682	18%	146/52	10/17	Secreted	Transport	1.52	0.0021	K.DLGEQHFK.G<br>K.GLVLIAFSQYLQK.C<br>K.CPYEEHIK.L
366	P02770	Serum albumin	6.09	70682	39%	257/52	21/29	Secreted	Transport	1.64	0.00016	R.FKDLGEQHFK.G<br>K.GLVLIAFSQYLQK.C<br>K.CPYEEHIK.L
370	P02770	Serum albumin	6.09	70682	13%	110/52	7/11	Secreted	Transport	1.66	0.0057	K.GLVLIAFSQYLQK.C<br>K.CPYEEHIK.L<br>K.LVQEVTDFAK.T
373	P02770	Serum albumin	6.09	70682	37%	238/52	20/29	Secreted	Transport	1.51	0.018	R.FKDLGEQHFK.G<br>K.DLGEQHFK.G<br>K.GLVLIAFSQYLQK.C
379	P02770	Serum albumin	6.09	70682	21%	170/52	12/21	Secreted	Transport	1.38	0.043	K.DLGEQHFK.G<br>K.GLVLIAFSQYLQK.C<br>K.CPYEEHIK.L<br>K.CPYEEHIK.L
1418	P02767	Transthyretin	5.77	15824	20%	70/52	4/22	Secreted	Thyroxine transport	1.77	0.00012	K.VLDAVR.G<br>K.TADGSWEPFASGK.T<br>K.FTEGVYR.V
591	Q5XIF6	Tubulin α-4A chain	4.95	50634	10%	62/52	4/8	Cytoplasm	Cytoskeleton	−1.66	0.034	K.EDAANNYAR.G<br>R.NLDIERPTYTNLNR.L<br>K.VGINYQPPTVVPGGDLAK.V

Table S1. *Cont.*

Master No.	Swiss-Prot No.	Protein Name	pI	MW	Cov. (%)	Score	Matched Peptides	Subcellular Location	Functional Ontology	IR/Ctrl Av. Ratio	IR/Ctrl *t*-Test	No. Match Peptides
686	P69897	Tubulin β-5 chain	4.78	50095	35%	231/52	18/25	Cytoplasm	Cytoskeleton	−1.54	0.0013	.MREIVHIQAGQCGNQIGAK.F + Oxidation (M)<br>R.EIVHIQAGQCGNQIGAK.F<br>R.ISVYYNEATGGK.Y
691	P69897	Tubulin β-5 chain	4.78	50095	10%	76/52	5/9	Cytoplasm	Cytoskeleton	−2.47	0.016	R.FPGQLNADLR.K<br>K.LAVNMVPFPR.L + Oxidation (M)<br>R.YLTVAAVFR.G
702	P69897	Tubulin β-5 chain	4.78	50095	18%	129/52	10/17	Cytoplasm	Cytoskeleton	−1.49	0.00023	R.AILVDLEPGTMDSVR.S + Oxidation (M)<br>K.IREEYPDR.I<br>R.IMNTFSVVPSPK.V + Oxidation (M)
690	P69897	Tubulin β-5 chain	4.78	50095	11%	86/52	8/17	Cytoplasm	Cytoskeleton	−1.94	0.00034	K.IREEYPDR.I R.IMNTFSVVPSPK.V + Oxidation (M)<br>R.FPGQLNADLR.K
1198	Q00981	Ubiquitin carboxyl-terminal hydrolase isozyme L1	5.14	25165	15%	57/52	3/6	Cytoplasm	Protein degradation	−1.86	0.0013	K.LGVAGQWR.F<br>K.NEAIQAAHDSVAQEGQCR.V<br>R.FSAVALCK.A
608	P04276	Vitamin D-binding protein	5.65	55106	13%	95/52	8/16	Secreted	Transport	1.39	0.0022	R.SLSLILYSR.K<br>R.VCSQYAAYGK.E<br>R.ELPEHTLK.I

Table S2. Alphabetical list of identified differentially expressed conjunctiva proteins between the IR injury and control groups after 2D-DIGE coupled with MALDI-TOF mass spectrometry analysis.

Master No.	Swiss-Prot No.	Protein Name	pI	MW	Cov. (%)	Score	Matched peptides	Subcellular location	Functional ontology	IR/Ctrl Av. Ratio	IR/Ctrl *t*-Test	No. Match Peptides
493	P17475	α-1-antiproteinase	5.7	46278	10%	75/52	5/9	Secreted	Protease inhibitor	1.34	0.039	K.RPFNPEHTR.D<br>R.DADFHVDK.S<br>R.SAILYFPK.L
700	Q63041	α-1-macroglobulin	6.46	168388	3%	64/52	6/14	Secreted	Heme degradation	2.23	0.00019	K.YGAATFTK.S<br>R.LLLQEVR.L<br>R.LADLPGNYITK.V
734	P07150	Annexin A1	6.97	39147	27%	131/52	8/17	Plasma membrane	Signal transduction/Ca2+ signaling	−1.49	0.035	M.AMVSEFLK.Q + Acetyl (Protein N-term) Oxidation (M)<br>K.GGPGSAVSPYPSFNPSSDVAALHK.A<br>K.TPAQFDADELR.A

Table S2. *Cont.*

Master No.	Swiss-Prot No.	Protein Name	pI	MW	Cov. (%)	Score	Matched peptides	Subcellular location	Functional ontology	IR/Ctrl Av. Ratio	IR/Ctrl *t*-Test	No. Match Peptides
792	P55260	Annexin A4	5.31	36168	16%	82/52	5/9	Plasma membrane	Signal transduction/Ca3+ signaling	−1.34	0.017	K.AASGFNATEDAQVLR.K<br>R.INQTYQQQYGR.S<br>R.VLVSLTAGGR.D
99	Q7TSU1	Brefeldin A-inhibited guanine nucleotide-exchange protein 2	6.08	204297	9%	61/52	14/37	Cytoplasm	Vescular transport	−1.54	0.03	.MQESQTK.S<br>M.QESQTKSMFVSR.A + Oxidation (M)<br>K.RPQHSQLRR.A
630	P09605	Creatine kinase S-type, mitochondrial	8.76	47811	22%	125/52	8/21	Mitochondria	Energy metabolism	1.64	0.018	K.LFPPSADYPDLR.K<br>R.HNGYDPR.L<br>K.ITHGQFDER.Y
117	Q7TQ16	Cytochrome b-c1 complex subunit 8	10.52	9843	58%	62/52	4/27	Mitochondria	Electron transport	−1.47	0.037	R.EFGNLTR.I<br>R.EFGNLTRIR.H<br>R.HVISYSLSPFEQR.A
112	P68101	Eukaryotic translation initiation factor 2 subunit 1	5.02	36371	17%	54/52	4/19	Cytoplasmic granule	Translation control	−1.5	0.026	.MPGLSCR.F + Oxidation (M)<br>K.FPEVEDVVMVNVR.S<br>R.SIAEMGAYVSLLEYNNIEGMILLSELSR.R
412	Q5J2D6	Gametogenetin-binding protein 1	5.78	41227	16%	61/52	7/26	Cytoplasm	Cell differentiation	−1.97	0.02	M.AAQARTPR.S<br>K.REPRPR.V<br>K.QLQKSAMTK.A + Gln->pyro-Glu (N-term Q)<br>Oxidation (M)
238	P20059	Hemopexin	7.58	52060	8%	56/52	4/10	Secreted	Heme transport	1.46	0.0085	K.NPVTSVDAAFR.G<br>R.GPDSVFLIK.E<br>R.FNPVTGEVPPR.Y
236	P20059	Hemopexin	7.58	52060	8%	58/52	4/9	Secreted	Heme transport	1.57	0.028	K.NPVTSVDAAFR.G<br>R.GPDSVFLIK.E<br>R.FNPVTGEVPPR.Y
940	Q6IFW6	Keratin, type I cytoskeletal 10	5.1	56699	17%	70/52	15/61	Cytoplasm	Cytoskeleton	2.07	0.005	K.VTMQNLNDR.L + Oxidation (M)<br>K.IKEWYEK.H<br>R.LAADDFR.L
596	Q6IFV3	Keratin, type I cytoskeletal 15	4.8	49011	26%	162/52	13/20	Cytoplasm	Cytoskeleton	−2.07	0.0015	M.ATTFLQTSSTFGSGSTR.G<br>+ Acetyl (Protein Nterm)<br>R.VGGGSFGGGSLYGGGGSR.S<br>K.VTMQNLNDR.L + Oxidation (M)
597	Q6IFV3	Keratin, type I cytoskeletal 15	4.8	49011	12%	60/52	5/10	Cytoplasm	Cytoskeleton	−2.32	0.00087	M.ATTFLQTSSTFGSGSTR.G + Acetyl (Protein Nterm)<br>R.VGGGSFGGGSLYGGGGSR.S<br>K.IRDWYQK.Q

Table S2. *Cont.*

Master No.	Swiss-Prot No.	Protein Name	pI	MW	Cov. (%)	Score	Matched peptides	Subcellular location	Functional ontology	IR/Ctrl Av. Ratio	IR/Ctrl *t*-Test	No. Match Peptides
572	Q6IFV3	Keratin, type I cytoskeletal 15	4.8	49011	13%	78/52	5/10	Cytoplasm	Cytoskeleton	−2.59	0.00056	M.ATTFLQTSSTFGSGSTR.G + Acetyl (Protein Nterm) R.VGGGSFGGGSLYGGGGSR.S K.VTMQNLNDR.L + Oxidation (M)
445	Q6IG00	Keratin, type II cytoskeletal 4	7.52	57973	6%	54/52	4/8	Cytoplasm	Cytoskeleton	−2.28	0.0031	R.CSSGGFGSR.S K.FASFIDK.V R.AQYEEIAR.K
401	Q6P6Q2	Keratin, type II cytoskeletal 5	7.6	61959	10%	90/52	6/13	Cytoplasm	Cytoskeleton	−1.64	0.024	R.QSSVSFR.S R.SFSAASAITPSVSR.T R.TTFSSVSR.S
406	Q6P6Q2	Keratin, type II cytoskeletal 5	7.6	61959	13%	115/52	8/14	Cytoplasm	Cytoskeleton	−1.74	0.0068	R.QSSVSFR.S R.SFSAASAITPSVSR.T R.TTFSSVSR.S
245	Q8R4Z9	Mitofusin-1	6.11	84649	7%	61/52	6/9	Mitochondria	Mitochondria morphology	1.5	0.044	.MAETVSPLK.H + Acetyl (Protein N-term) K.HFVLAKK.A K.LAVIGEVLSRR.H
378	Q8K5B3	Multiple coagulation factor deficiency protein 2 homolog	4.61	16252	16%	52/52	3/6	Cytoplasm	Vescular transport	1.42	0.00042	.MASLQLLR.G + Acetyl (Protein N-term) M.ASLQLLR.G + Acetyl (Protein N-term) K.NNDGYIDYAEFAKSLQ.-
766	P52873	Pyruvate carboxylase, mitochondrial	6.34	130436	8%	72/52	10/25	Mitochondria	Gluconeogenesis	1.55	0.014	R.GEIAIRVFR.A R.ACTELGIR.T R.LTSDSVKLAK.Q
119	Q6AYT0	Quinone oxidoreductase	8.43	35295	16%	58/52	5/45	Mitochondria	Electron transport	−1.46	0.032	R.AIRVFEFGGPEVLK.L K.EANYIDK.I K.NLSNDLKLLSCGGR.V
169	P12346	Serotransferrin	7.14	78512	14%	147/52	11/20	Secreted	Iron transport	1.49	0.015	K.TVLPADGPR.L K.GTDFQLNQLQGK.K K.SCHTGLGR.S
217	P12346	Serotransferrin	7.14	78512	17%	164/52	12/24	Secreted	Iron transport	1.53	0.042	K.TVLPADGPR.L R.DGGGDVAFVK.H K.HTTIFEVLPQK.A
223	P12346	Serotransferrin	7.14	78512	26%	208/52	18/50	Secreted	Iron transport	1.56	0.037	K.WCAVSEHENTK.C K.TVLPADGPR.L K.SCHTGLGR.S
212	P02770	Serum albumin	6.09	70682	8%	64/52	5/12	Secreted	Transport	1.48	0.0051	K.DLGEQHFK.G K.LVQEVTDFAK.T K.SIHTLFGDK.L

Table S2. *Cont.*

Master No.	Swiss-Prot No.	Protein Name	pI	MW	Cov. (%)	Score	Matched peptides	Subcellular location	Functional ontology	IR/Ctrl Av. Ratio	IR/Ctrl *t*-Test	No. Match Peptides
302	P02770	Serum albumin	6.09	70682	26%	127/52	15/36	Secreted	Transport	1.57	0.049	R.FKDLGEQHFK.G<br>K.DLGEQHFK.G<br>K.GLVLIAFSQYLQK.C
303	P02770	Serum albumin	6.09	70682	20%	134/52	11/29	Secreted	Transport	1.58	0.045	K.DLGEQHFK.G<br>K.GLVLIAFSQYLQK.C<br>K.LVQEVTDFAK.T
312	P02770	Serum albumin	6.09	70682	33%	194/52	21/43	Secreted	Transport	1.53	0.039	K.SEIAHRFK.D<br>R.FKDLGEQHFK.G<br>K.DLGEQHFK.G
335	P02770	Serum albumin	6.09	70682	28%	167/52	15/44	Secreted	Transport	1.63	0.026	K.DLGEQHFK.G K.GLVLIAFSQYLQK.C<br>K.CPYEEHIK.L
571	Q68FW7	Threonine--tRNA ligase, mitochondrial	8.07	82647	6%	56/52	7/19	Mitochondria	Translation control	−2.16	0.0014	.MGLCLR.W + Acetyl (Protein N-term) Oxidation (M)<br>.MGLCLRWR.R + Oxidation (M)<br>R.STELPTLER.I
482	P04276	Vitamin D-binding protein	5.65	55106	7%	55/52	6/10	Secreted	Transport	1.36	0.021	R.SLSLILYSR.K<br>R.RTQVPEVFLSK.V<br>R.TQVPEVFLSK.V
118	Q71LX6	Xin actin-binding repeat-containing protein 2	5.74	375861	7%	59/52	13/42	Cytoplasm	Cytoskeleton	−1.47	0.033	K.DLSSERENLEWDEILK.G<br>K.ENQEGDGLVKTVTDIQGGDVR<br>K.ETLEDLYSQRVVEAPGIIIEAD

Table S3. Alphabetical list of identified differentially expressed uvea proteins between the IR injury and control groups after 2D-DIGE coupled with MALDI-TOF mass spectrometry analysis.

Master No.	Swiss-Prot No.	Protein Name	pI	MW	Cov. (%)	Score	Matched Peptides	Subcellular Location	Functional Ontology	IR/Ctrl Av. Ratio	IR/Ctrl *t*-Test	No. Match Peptides
561	P68035	Actin, α cardiac muscle 1	5.2	42334	32%	139/52	12/32	Cytoplasm	Cytoskeleton	4.48	0.011	K.AGFAGDDAPR.A R.AVFPSIVGRPR.H R.HQGVMVGMGQK.D + Oxidation (M)
546	P68035	Actin, α cardiac muscle 1	5.2	42334	18%	91/52	6/14	Cytoplasm	Cytoskeleton	1.57	0.038	K.AGFAGDDAPR.A K.IWHHTFYNELR.V R.DLTDYLMK.I + Oxidation (M)
564	P68035	Actin, α cardiac muscle 1	5.2	42334	18%	90/52	6/15	Cytoplasm	Cytoskeleton	1.88	0.001	K.AGFAGDDAPR.A K.IWHHTFYNELR.V R.DLTDYLMK.I + Oxidation (M)
507	P68136	Actin, α skeletal muscle	5.2	42366	34%	143/52	13/30	Cytoplasm	Cytoskeleton	3.02	0.04	K.AGFAGDDAPR.A R.AVFPSIVGRPR.H R.HQGVMVGMGQK.D + Oxidation (M)
1204	P23928	α-crystallin B chain	6.8	20076	42%	117/52	10/32	Cytoplasm	Protein folding	3.09	0.033	.MDIAIHHPWIR.R + Acetyl (Protein N-term) .MDIAIHHPWIR.R + Acetyl (Protein N-term) Oxidation (M) R.RPFFPFHSPSR.L
413	P15999	ATP synthase subunit α, mitochondrial	9.2	59831	22%	103/52	13/26	Mitochondria	Energy metabolism	1.59	0.039	R.ILGADTSVDLEETGR.V R.VLSIGDGIAR.V R.TGAIVDVPVGDELLGR.V
454	P10719	ATP synthase subunit β, mitochondrial	5.2	56318	33%	158/52	16/30	Mitochondria	Energy metabolism	1.65	0.021	R.TIAMDGTEGLVR.G R.TIAMDGTEGLVR.G + Oxidation (M) K.VLDSGAPIKIPVGPETLGR.I
484	P15429	β-enolase	7.1	47326	17%	121/52	8/16	Cytoplasm	Glycolysis	6.7	0.0011	R.GNPTVEVDLHTAK.G K.AVEHINK.T K.TLGPALLEK.K
1276	P62161	Calmodulin	4.1	16827	24%	52/52	5/17	Cytoplasm	Siganl transduction / Ca regulation	-2.64	0.013	K.EAFSLFDK.D K.EAFSLFDKDGDGTITTK.E K.ELGTVMR.S
1305	B0BNA5	Coactosin-like protein	5.3	16036	27%	81/52	5/17	Cytoplasm	Cytoskeleton regulation	-1.38	0.034	R.AAYNLVR.D R.FTTGDAMSK.R + Oxidation (M) K.EVVQNFAK.E

Table S3. *Cont.*

Master No.	Swiss-Prot No.	Protein Name	pI	MW	Cov. (%)	Score	Matched Peptides	Subcellular Location	Functional Ontology	IR/Ctrl Av. Ratio	IR/Ctrl *t*-Test	No. Match Peptides
619	P00564	Creatine kinase M-type	6.6	43246	20%	108/52	7/16	Cytoplasm	Energy metabolism	4.59	0.00061	M.PFGNTHNK.F<br>K.DLFDPIIQDR.H<br>K.GGDDLDPNYVLSSR.V
574	P00564	Creatine kinase M-type	6.6	43246	18%	91/52	8/17	Cytoplasm	Energy metabolism	2.58	0.02	M.PFGNTHNK.F<br>M.PFGNTHNKFK.L + Acetyl (Protein N-term)<br>K.VLTPDLYNK.L
621	P00564	Creatine kinase M-type	6.6	43246	24%	143/52	9/22	Cytoplasm	Energy metabolism	17.11	9.1×10^{-5}	M.PFGNTHNK.F<br>K.HNNHMAK.V<br>K.VLTPDLYNK.L
622	P00564	Creatine kinase M-type	6.6	43246	19%	115/52	8/18	Cytoplasm	Energy metabolism	5.83	0.012	M.PFGNTHNK.F<br>K.VLTPDLYNK.L<br>K.DLFDPIIQDR.H
547	P09605	Creatine kinase S-type, mitochondrial	8.8	47811	22%	128/52	8/18	Cytoplasm	Energy metabolism	6.48	0.0019	K.LFPPSADYPDLR.K<br>R.HNGYDPR.L<br>K.ITHGQFDER.Y
1317	P12075	Cytochrome c oxidase subunit 5B, mitochondrial	7.7	14191	20%	52/52	3/12	Mitochondria	Electron transport	1.57	0.007	R.EIMIAAQR.G + Oxidation (M)<br>R.GLDPYNMLPPK.A + Oxidation (M)<br>K.LVPYQMVH.- + Oxidation (M)
1271	Q7M0E3	Destrin	8.2	18807	23%	64/52	5/12	Cytoplasm	Cytoskeleton regulation	-1.51	0.023	M.ASGVQVADEVCR.I + Acetyl (Protein N-term)<br>R.IFYDMK.V<br>R.IFYDMK.V + Oxidation (M)
230	P08461	Dihydrolipoyllysine-residue acetyltransferase component of pyruvate dehydrogenase complex, mitochondrial	8.8	67637	12%	104/52	7/11	Mitochondria	TCA cycle	1.77	0.02	K.VPLPSLSPTMQAGTIAR.W + Oxidation (M)<br>K.ILVPEGTR.D<br>R.VFVSPLAK.K
787	Q6AXU4	E3 ubiquitin-protein ligase RNF181	5.7	19674	20%	57/52	4/18	Cytoplasm	Protein degradation	8.76	0.0048	R.NNMLLELAR.R<br>K.AVVESLPR.T<br>R.QQQQHR.L
925	P52555	Endoplasmic reticulum resident protein 29	6.2	28614	17%	69/52	4/9	ER	Vescular transport	-1.53	0.0098	K.GALPLDTVTFYK.V<br>K.FDTQYPYGEK.Q<br>R.DGDFENPVPYSGAVK.V
1330	P11762	Galectin-1	5.1	15189	45%	97/52	7/28	Plasma membrane	Cell growth	-1.65	0.0079	R.GELAPDAK.S<br>K.SFVLNLGK.D<br>K.DSNNLCLHFNPR.F

Table S3. *Cont.*

Master No.	Swiss-Prot No.	Protein Name	pI	MW	Cov. (%)	Score	Matched Peptides	Subcellular Location	Functional Ontology	IR/Ctrl Av. Ratio	IR/Ctrl *t*-Test	No. Match Peptides
731	P04797	Glyceraldehyde-3-phosphate dehydrogenase	8.1	36090	20%	101/52	6/15	Cytoplasm	Glycolysis	1.93	0.011	K.VGVNGFGR.I R.GAAQNIIPASTGAAK.A K.LTGMAFR.V + Oxidation (M)
747	P04797	Glyceraldehyde-3-phosphate dehydrogenase	8.1	36090	20%	91/52	6/24	Cytoplasm	Glycolysis	1.8	0.023	K.VGVNGFGR.I R.GAAQNIIPASTGAAK.A K.LTGMAFR.V + Oxidation (M)
768	P04797	Glyceraldehyde-3-phosphate dehydrogenase	8.1	36090	19%	105/52	6/18	Cytoplasm	Glycolysis	1.54	0.029	K.VGVNGFGR.I R.GAAQNIIPASTGAAK.A K.LTGMAFR.V + Oxidation (M)
751	P04797	Glyceraldehyde-3-phosphate dehydrogenase	8.1	36090	27%	118/52	7/18	Cytoplasm	Glycolysis	1.63	0.03	K.VGVNGFGR.I R.VIISAPSADAPMFVMGVNHEK.Y + 2 Oxidation (M) R.GAAQNIIPASTGAAK.A
784	A7VJC2	Heterogeneous nuclear ribonucleoproteins A2/B1	9	37512	17%	92/52	6/14	Nucleus	Transcription control	-1.62	2.7×10^{-5}	R.NYYEQWGK.L R.DYFEEYGK.I K.IDTIEIITDR.Q
573	P21213	Histidine ammonia-lyase	6.1	72923	9%	53/52	4/13	Cytoplasm	Amino acid catabolism	1.45	0.047	K.NKPDNGGFTSVDEVR.F R.VQDAYTLR.C K.DIITTELNSATDNPMVFASR. + Oxidation (M)
670	Q99NA5	Isocitrate dehydrogenase (NAD) subunit α, mitochondrial	6.5	40044	20%	107/52	7/14	Mitochondria	TCA cycle	1.79	0.00098	K.APIQWEER.N K.TPIAAGHPSMNLLLR.K + Oxidation (M) K.TPYTDVNIVTIR.E
563	Q63560	Microtubule-associated protein 6	9.5	100651	4%	52/52	5/7	Cytoplasm	Cytoskeleton regulation	1.76	0.015	M.AWPCITRACCIAR.F K.DKQVASGQAAK.K K.QVASGQAAK.K + Gln->pyro-Glu (N-term Q)
1149	P02600	Myosin light chain 1/3, skeletal muscle isoform	5	20781	45%	105/52	8/48	Cytoplasm	Cytoskeleton	36.36	0.016	K.KPAAAAPAPAPAPAPAPAKPK.E K.EAFLLFDR.T K.ITLSQVGDVLR.A
1163	P02600	Myosin light chain 1/3, skeletal muscle isoform	5	20781	45%	93/52	7/42	Cytoplasm	Cytoskeleton	24.77	0.0049	K.KPAAAAPAPAPAPAPAPAKPK.E K.EAFLLFDR.T K.ITLSQVGDVLR.A

Table S3. *Cont.*

Master No.	Swiss-Prot No.	Protein Name	pI	MW	Cov. (%)	Score	Matched Peptides	Subcellular Location	Functional Ontology	IR/Ctrl Av. Ratio	IR/Ctrl *t*-Test	No. Match Peptides
1329	P02600	Myosin light chain 1/3, skeletal muscle isoform	5	20781	28%	68/52	5/29	Cytoplasm	Cytoskeleton	4.48	0.03	K.EAFLLFDR.T<br>K.ITLSQVGDVLR.A<br>R.ALGTNPTNAEVK.K
1258	P04466	Myosin regulatory light chain 2, skeletal muscle isoform	4.8	19071	39%	107/52	7/17	Cytoplasm	Cytoskeleton	4.22	0.023	K.EAFTVIDQNR.D<br>R.DTFAAMGR.L<br>R.DTFAAMGR.L + Oxidation (M)
1273	P04466	Myosin regulatory light chain 2, skeletal muscle isoform	4.8	19071	39%	103/52	7/20	Cytoplasm	Cytoskeleton	25.7	6.7×10^{-5}	K.EAFTVIDQNR.D<br>R.DTFAAMGR.L<br>R.DTFAAMGR.L + Oxidation (M)
1331	Q6AXQ4	Myotubularin	8	69878	7%	52/52	4/10	Cytoplasm	Lipid catabolism	1.85	0.00058	M.ASSSASDCDAHPVER.E + Acetyl (Protein N-term)<br>R.GENSYGLDITCK.D<br>K.LLLTGAIR.V
1314	P02625	Parvalbumin alpha	5	11918	54%	134/52	9/20	Nucleus	Siganl transduction / Ca regulation	10.32	0.00049	M.SMTDLLSAEDIKK.A + Acetyl (Protein N-term); Oxidation (M)<br>K.AIGAFTAADSFDHK.K<br>K.AIGAFTAADSFDHKK.F
1284	P10111	Peptidyl-prolyl cis-trans isomerase A	8.3	18091	26%	68/52	4/14	Cytoplasm	Protein folding	-1.82	0.041	M.VNPTVFFDITADGEPLGR.V<br>R.VCFELFADK.V<br>K.FEDENFILK.H
1193	P31044	Phosphatidylethanolamine-binding protein 1	5.5	20902	28%	64/52	4/9	Plasma membrane	Siganl transduction	-1.37	0.043	R.VDYGGVTVDELGK.V<br>K.LYTLVLTDPDAPSR.K<br>K.GNDISSGTVLSEYVGSGPPK.D
236	P20961	Plasminogen activator inhibitor 1	6.2	45038	10%	54/52	4/7	Secreted	Coagulation	1.42	0.011	.MQMSSALTCLTLGLVLVFGK.G + Acetyl (Protein N-term)<br>K.LFRTTVK.Q<br>K.SNMTRLPR.L
1311	P19356	Porphobilinogen deaminase	6.2	39622	8%	56/52	5/9	Cytoplasm	Heme biosynthesis	-1.87	0.0068	K.SAVGTSSLRR.V<br>R.RVAQLQR.K<br>R.VAQLQRK.F
317	P11598	Protein disulfide-isomerase A3	5.9	57044	21%	152/52	12/22	Cytoplasm	Protein folding	-1.47	0.025	R.LAPEYEAAATR.L<br>K.YGVSGYPTLK.I<br>R.DGEEAGAYDGPR.T

Table S3. *Cont.*

Master No.	Swiss-Prot No.	Protein Name	pI	MW	Cov. (%)	Score	Matched Peptides	Subcellular Location	Functional Ontology	IR/Ctrl Av. Ratio	IR/Ctrl *t*-Test	No. Match Peptides
818	P49432	Pyruvate dehydrogenase E1 component subunit β, mitochondrial	6.2	39299	22%	128/52	7/22	Mitochondria	TCA cycle	1.55	0.014	K.TYYMSAGLQPVPIVFR.G + Oxidation (M)<br>K.DFLIPIGK.A<br>K.EGIECEVINLR.T
1256	Q9WUW8	Sulfotransferase 1C2	8.2	34961	5%	52/52	3/5	Cytoplasm	Sulfo transfer	11.82	0.0063	.MALAPELSR.Q + Acetyl (Protein N-term); Oxidation (M)<br>M.ALAPELSR.Q<br>K.ANAMPAPR.I
548	Q9WUW8	Sulfotransferase 1C2	8.2	34961	5%	52/52	3/5	Cytoplasm	Sulfo transfer	2.24	0.031	.MALAPELSR.Q + Acetyl (Protein N-term); Oxidation (M)<br>M.ALAPELSR.Q<br>K.ANAMPAPR.I
777	P09495	Tropomyosin α-4 chain	4.7	28549	19%	53/52	7/24	Cytoplasm	Cytoskeleton	5.78	0.015	.MAGLNSLEAVKR.K + Acetyl (Protein N-term)<br>.MAGLNSLEAVKR.K + Acetyl (Protein N-term); Oxidation (M)<br>K.HIAEEADR.K
745	P58775	Tropomyosin β chain	4.7	32931	28%	101/52	13/40	Cytoplasm	Cytoskeleton	5.25	0.00085	K.LDKENAIDR.A<br>K.KATDAEADVASLNR.R<br>K.ATDAEADVASLNR.R

Table S4. Alphabetical list of identified differentially expressed sclera proteins between the IR injury and control groups after 2D-DIGE coupled with MALDI-TOF mass spectrometry analysis.

Master No.	Swiss-Prot No.	Protein Name	pI	MW	Cov. (%)	Score	Matched Peptides	Subcellular Location	Functional Ontology	IR/Ctrl Av. Ratio	IR/Ctrl *t*-Test	No. Match Peptides
592	P00564	Creatine kinase M-type	6.6	43246	13%	61/52	4/10	Cytoplasm	Energy metabolism	−2.44	0.0016	K.GGDDLDPNYVLSSR.V<br>K.GYTLPPHCSR.G<br>K.FEEILTR.L
219	P63018	Heat shock cognate 71 kDa protein	5.4	71055	10%	62/52	5/15	Cytoplasm	Protein folding	−1.37	0.046	R.TTPSYVAFTDTER.L<br>K.DAGTIAGLNVLR.I<br>K.STAGDTHLGGEDFDNR.M
476	Q6AXT5	Ras-related protein Rab-21	8.1	24547	18%	60/52	3/6	Cytoplasm	Vescular transport	−1.3	0.1	.MAAAGGGAAAAAGR.A + Acetyl (Protein N-term)<br>K.VVLLGEGCVGK.T<br>R.HVSIQEAESYAESVGAK.H
239	P02770	Serum albumin	6.1	70682	12%	85/52	6/13	Secreted	Transport	1.38	0.036	K.GLVLIAFSQYLQK.C<br>K.LVQEVTDFAK.T<br>K.SIHTLFGDK.L

Table S5. Alphabetical list of identified differentially expressed retina proteins between the IR injury and control groups after 2D-DIGE coupled with MALDI-TOF mass spectrometry analysis.

Master No.	Swiss-Prot No.	Protein name	pI	MW	Cov. (%)	Score	Matched Peptides	Subcellular Location	Functional Ontology	IR/Ctrl Av. Ratio	IR/Ctrl *t*-Test	No. Match Peptides
354	P13233	2',3'-cyclic-nucleotide 3'-phosphodiesterase	9	47638	9%	54/52	4/10	Plasma membrane	RNA catabolism	−1.43	0.032	K.MVSADAYK.I<br>K.NQWQLSLDDLK.K<br>K.AIFTGYYGK.G
301	P11883	Aldehyde dehydrogenase, dimeric NADP-preferring	6.3	50706	23%	101/52	9/23	Cytoplasm	Redox reguation	−4.29	0.0002	R.IQQLEALQR.M<br>R.FDHIMYTGSTAVGK.I + Oxidation (M)<br>K.HLTPVTLELGGK.S
339	P11883	Aldehyde dehydrogenase, dimeric NADP-preferring	6.3	50706	35%	201/52	19/36	Cytoplasm	Redox reguation	−9.97	0.00047	M.SSISDTVKR.A + Acetyl (Protein N-term)<br>R.IQQLEALQR.M<br>K.SISGALASDLGK.N
225	P47942	Dihydropyrimidinase-related protein 2	6	62638	12%	75/52	6/18	Cytoplasm	Neuronal development	1.95	0.0042	R.MVIPGGIDVHTR.F + Oxidation (M)<br>R.SITIANQTNCPLYVTK.V<br>K.SAAEVIAQAR.K

Table S5. *Cont.*

Master No.	Swiss-Prot No.	Protein name	pI	MW	Cov. (%)	Score	Matched Peptides	Subcellular Location	Functional Ontology	IR/Ctrl Av. Ratio	IR/Ctrl *t*-Test	No. Match Peptides
267	P47942	Dihydropyrimidinase-related protein 2	6	62638	15%	118/52	8/15	Cytoplasm	Neuronal development	5.02	1.00×10^{-5}	R.MVIPGGIDVHTR.F<br>R.MVIPGGIDVHTR.F + Oxidation (M)<br>R.SITIANQTNCPLYVTK.V
818	P04797	Glyceraldehyde-3-phosphate dehydrogenase	8.1	36090	31%	117/52	10/32	Cytoplasm	Glycolysis	−1.34	0.035	K.VGVNGFGR.I<br>R.VIISAPSADAPMFVMGVNHEK.Y<br>R.VIISAPSADAPMFVMGVNHEK.Y + Oxidation (M)
321	Q6P6Q2	Keratin, type II cytoskeletal 5	7.6	61959	11%	93/52	7/15	Cytoplasm	Cytoskeleton	−1.9	0.039	R.VSLGGAYGAGGYGSR.S<br>R.ISFSSGGGSFR.N<br>K.FASFIDK.V
347	Q6P6Q2	Keratin, type II cytoskeletal 5	7.6	61959	18%	120/52	9/22	Cytoplasm	Cytoskeleton	−1.35	0.016	R.SFSAASAITPSVSR.T<br>R.TTFSSVSR.S<br>R.VSLGGAYGAGGYGSR.S
322	Q7TN44	Mas-related G-protein coupled receptor member B5	8.9	40897	10%	54/52	4/7	Plasma membrane	Signal transduction	−2.54	0.0025	R.LLGFHMHR.Y<br>K.IICGSHR.I<br>R.QSLKLLLQR.A
416	P19356	Porphobilinogen deaminase	6.2	39622	10%	60/52	6/14	Cytoplasm	Heme biosynthesis	−1.95	0.039	K.SAVGTSSLRR.V<br>R.RVAQLQR.K<br>R.VAQLQRK.F
281	P11598	Protein disulfide-isomerase A3	5.9	57044	11%	79/52	7/14	Cytoplasm	Protein folding	2.94	0.00017	R.LAPEYEAAATR.L<br>K.QAGPASVPLR.T + Gln->pyro-Glu (N-term Q)<br>K.QAGPASVPLR.T
890	Q8VD52	Pyridoxal phosphate phosphatase	5.4	33493	26%	110/52	6/11	Cytoplasm	Vitamin B6 catabolism	1.83	0.017	R.IVPGAPELLQR.L<br>K.ATLFVSNNSR.R<br>R.AEELFSSAVCAAR.L
350	P11980	Pyruvate kinase PKM	6.6	58294	27%	164/52	14/33	Cytoplasm	Glycolysis	−2.06	0.00028	R.LDIDSAPITAR.N<br>R.NTGIICTIGPASR.S<br>R.LNFSHGTHEYHAETIK.N

Table S5. *Cont.*

Master No.	Swiss-Prot No.	Protein name	pI	MW	Cov. (%)	Score	Matched Peptides	Subcellular Location	Functional Ontology	IR/Ctrl Av. Ratio	IR/Ctrl *t*-Test	No. Match Peptides
164	P12346	Serotransferrin	7.1	78512	11%	120/52	8/13	Secreted	Iron transport	2.22	0.00024	K.TVLPADGPR.L<br>R.IPSHAVVAR.N<br>K.DSAFGLLR.V
176	P12346	Serotransferrin	7.1	78512	20%	215/52	15/22	Secreted	Iron transport	2.48	4.3×10^{-5}	K.TVLPADGPR.L<br>K.SCHTGLGR.S<br>R.DGGGDVAFVK.H
163	Q9QUL6	Vesicle-fusing ATPase	6.6	83170	13%	130/52	11/20	Cytoplasm	Vescular transport	1.43	0.015	K.DYQSGQHVMVR.T + Oxidation (M)<br>K.YIFTLR.T<br>K.EFSDIFR.R
